# Saltatory Conduction along Myelinated Axons Involves a Periaxonal Nanocircuit

**DOI:** 10.1016/j.cell.2019.11.039

**Published:** 2020-01-23

**Authors:** Charles C.H. Cohen, Marko A. Popovic, Jan Klooster, Marie-Theres Weil, Wiebke Möbius, Klaus-Armin Nave, Maarten H.P. Kole

**Affiliations:** 1 Department of Axonal Signalling, Netherlands Institute for Neuroscience, Royal Netherlands Academy for Arts and Sciences, Meibergdreef 47, 1105 BA Amsterdam, the Netherlands; 2 Cell Biology, Neurobiology and Biophysics, Department of Biology, Faculty of Science, Utrecht University, Padualaan 8, 3584 CH Utrecht, the Netherlands; 3 Department of Neurogenetics, Max-Planck-Institute for Experimental Medicine, Göttingen, Hermann-Rein-Strasse 3, 37075 Göttingen, Germany; 4 Center for Nanoscale Microscopy and Molecular Physiology of the Brain (CNMPB), Göttingen, Germany

**Keywords:** myelin, axon, action potential, saltatory conduction, periaxonal space, internode, computational modelling, circuit, single cable, double cable

## Abstract

The propagation of electrical impulses along axons is highly accelerated by the myelin sheath and produces saltating or “jumping” action potentials across internodes, from one node of Ranvier to the next. The underlying electrical circuit, as well as the existence and role of submyelin conduction in saltatory conduction remain, however, elusive. Here, we made patch-clamp and high-speed voltage-calibrated optical recordings of potentials across the nodal and internodal axolemma of myelinated neocortical pyramidal axons combined with electron microscopy and experimentally constrained cable modeling. Our results reveal a nanoscale yet conductive periaxonal space, incompletely sealed at the paranodes, which separates the potentials across the low-capacitance myelin sheath and internodal axolemma. The emerging double-cable model reproduces the recorded evolution of voltage waveforms across nodes and internodes, including rapid nodal potentials traveling in advance of attenuated waves in the internodal axolemma, revealing a mechanism for saltation across time and space.

## Highlights

• Cable modeling reveals myelin and submyelin parameters consistent with EM
• The periaxonal space is conductive and partially sealed at the paranodes
• Optically recorded *V*_m_ confirms the separation of axon and myelin circuits
• Double-cable internodes produce both temporal and amplitude saltation in *V*_m_

## Graphical Abstract

**Figure:**
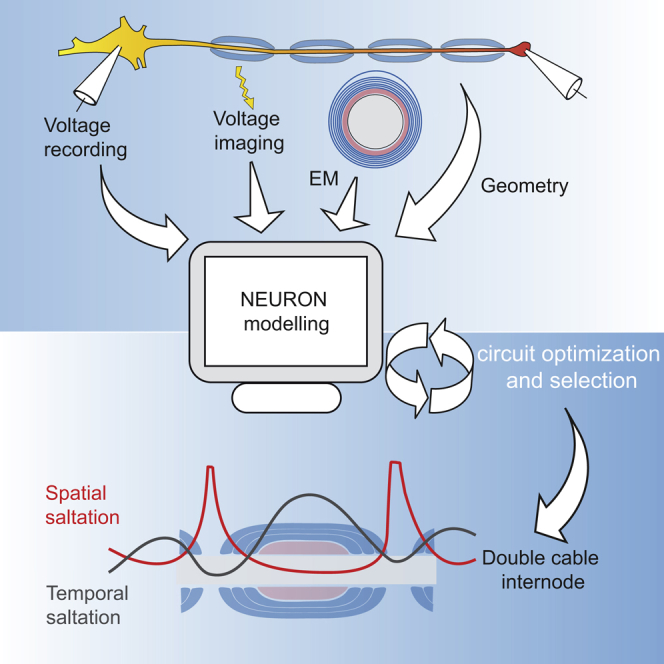

## Teaser

Patch-clamp recording and computational modeling combined with high-speed voltage-calibrated optical recordings and EM analysis reveal a second longitudinal conducting pathway formed by the periaxonal and paranodal submyelin spaces that are integral to reproducing the spatiotemporal profile of action potential saltation.

## Introduction

Vertebrate axons are ensheathed by multiple compacted myelin membranes spirally wrapped around the axolemma between the nodes of Ranvier, forming the anatomical basis for the rapid and saltatory conduction of electrical impulses in peripheral and central nervous systems (Hartline and Colman, 2007). In recent years, many important insights have emerged regarding the cellular and molecular organization of myelination, including the neuro-glial interactions regulating sheath formation and activity or experience-dependent regulation of myelination (Fields, 2015, McKenzie et al., 2014, Nave and Werner, 2014, Snaidero et al., 2014). However, in part due to the physical limitation in accessing the axolemma beneath the myelin sheath, our understanding of how myelination accelerates action potential (AP) propagation remains incomplete.

The common model for saltatory conduction is based on the seminal electrophysiological recordings of frog sciatic nerve by Tasaki, 1939, Tasaki, 1955 and Huxley and Stämpfli (1949). In the equivalent circuit representation, the axon and myelin sheath form one tightly combined membrane without intermediary conducting pathways, here referred to as “single cable” (SC). Although the view that myelin insulates internodal axolemma is widely accepted, alternative models that include submyelin or intramyelin conduction pathways have previously been proposed. Based on sharp-electrode intracellular recordings (Barrett and Barrett, 1982, Blight and Someya, 1985, Funch and Faber, 1984) and computer simulations (Arancibia-Cárcamo et al., 2017, Blight, 1985, Dimitrov, 2005, Gow and Devaux, 2008, McIntyre et al., 2002, Richardson et al., 2000, Stephanova and Bostock, 1995, Young et al., 2013), it is thought that axial conduction may occur between the myelin sheath and axon membrane, in the fluid-filled periaxonal and paranodal spaces, forming thereby an equivalent circuit referred to as “double cable” (DC). Submyelin current flow via the paranodal domains and within the periaxonal space would be consistent with tracer and electron microscopy (EM) studies indicating a continuity between the extracellular medium and submyelin or mesaxonal spaces (Hirano and Dembitzer, 1969, Rosenbluth et al., 2013, Shroff et al., 2011). However, given the lack of experimental data for potentials beneath or across the myelin sheath, the evidence for or against the involvement of submyelin conduction remains contentious. Furthermore, optical recordings of APs using voltage-sensitive dyes (VSDs) reveals a strikingly complex gradual pattern of onset latencies along the internodes and nodes (Popovic et al., 2011, Stuart and Palmer, 2006), which remains to be explained.

Detailed insight into electrical membrane properties of neurons and glia can be achieved by patch-clamp recordings combined with morphology-constrained passive cable modeling of voltage transients (Chan et al., 2013, Major et al., 1994, Nörenberg et al., 2010, Rall et al., 1992, Rapp et al., 1994, Roth and Häusser, 2001, Schmidt-Hieber et al., 2007). Here, we hypothesized that such an approach may yield direct insights into the intra-internodal circuit pathways in myelinated axons. Using computational modeling in combination with high-speed voltage-calibrated optical recordings of the axolemma, as well as EM analysis, we find evidence for a second longitudinal conducting pathway formed by the periaxonal and paranodal submyelin spaces. The resulting DC model reveals that a capacitance-lowering myelin sheath combined with a conducting submyelin layer are integral to reproduce the spatiotemporal profile of AP saltation.

## Results

### A DC Circuit Is Necessary to Account for Axonal Voltage Transients

**Figure 1:**
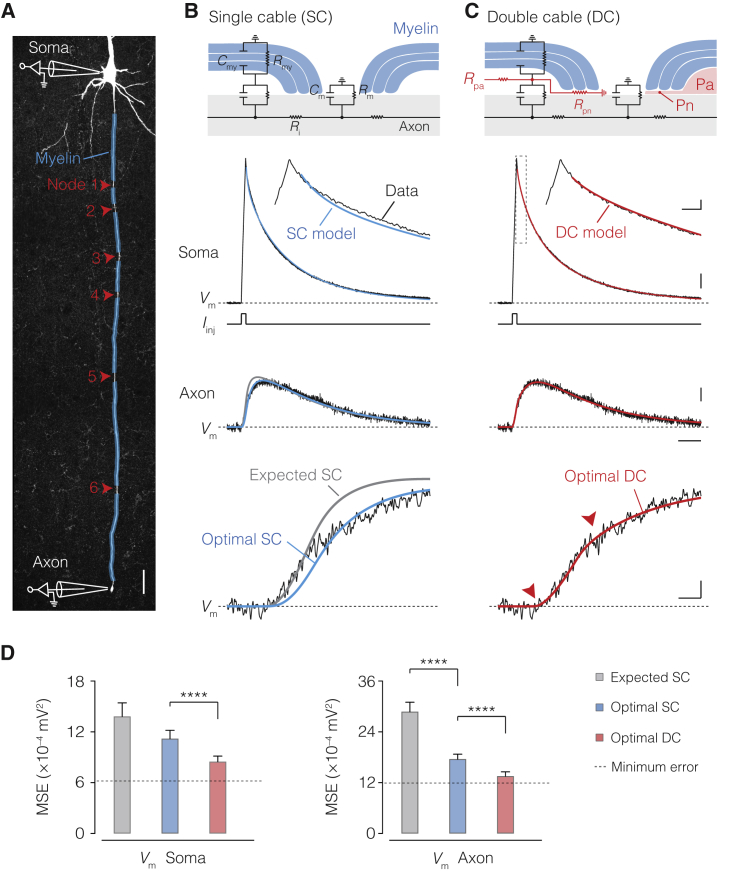
Cable Modeling Reveals a Submyelin Conduction Pathway (A) Confocal image of a L5 axon with illustration of patch-clamp recording sites. Red arrows indicate node locations, transparent blue myelin sheaths. Scale bar, 50 μm. (B) Top to bottom, equivalent circuit of the SC model of internodes. Middle traces, optimized SC solutions (blue) overlaid with somatic and axonal voltage transients (black, traces of cell #5) evoked with a brief somatic current injection. For comparison, a SC model of myelin with an expected 20 myelin membranes (*R*_my_ = 20 × *R*_m_, *C*_my_ = 0.05 × *C*_m_, gray). Bottom, rising phase of axonal *V*_m_ with optimal SC (blue) or expected SC solutions (gray). (C) Top to bottom, schematic of the equivalent DC internodal circuit, with added periaxonal (Pa) and paranodal (Pn) axial resistances (red). Middle traces, same recording as in B overlaid with optimized DC solutions (red). Note the improved fit of the DC model to the inflection points (red arrows). Scale bars, top to bottom, 0.1 mV and 0.5 ms, 1 mV, 0.2 mV and 10 ms, 0.1 mV and 1 ms. (D) Population optimization error (mean squared error, MSE) for the expected (gray) and optimal SC (blue) and DC (red). The DC model improved solutions for both somatic and axonal recordings (Friedman test with Dunn’s correction p < 0.0001; n = 6 neurons with 8 voltage responses optimized from 8 current injections each). Column and error bars represent mean ± SEM. See also Figures S1, S2, S3, and S4 and Tables S1–S4.

**Figure S1:**
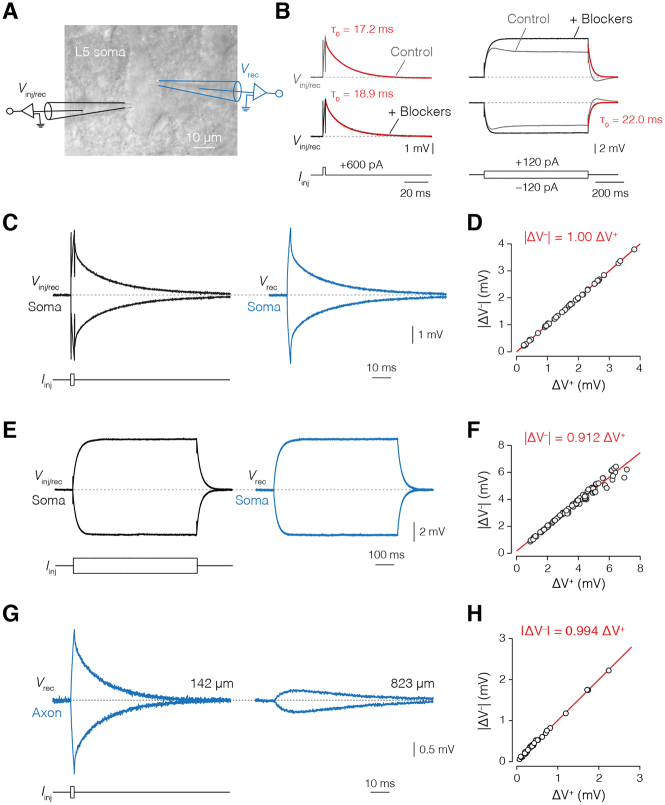
Linearity of Somatic and Axonal Voltage Responses, Related to Figure 1 (A), Schematic of the injecting/recording (*V*_inj/rec_) and recording-only (*V*_rec_) patch-clamp electrodes at the soma of a thick-tufted L5 pyramidal neuron overlaid with brightfield image. (B), Comparison of voltage transients to brief (left) and long (right) current injections (2 ms, 600 pA and 700 ms, 120 pA, respectively) from the *V*_inj/rec_ electrode at the soma in control conditions (gray) or after the addition of a solution with conductance blockers (black). Note the uniform exponential decay observed in the ensuing steady-state responses in blocker (black) versus previous non-blocker (gray) conditions, consistent across recordings. (C), example traces for voltage recordings of passive transients used for cable modeling in blocker conditions where *V*_Rec_ (blue) yields a near-identical result to *V*_inj/rec_ (black). See STAR Methods for analysis of the parameter differences between these. (D), stepwise linearity of short-pulse recordings at all somatic recording sites (n = 19 cells) showing complete linearity of voltage responses (|ΔV^–^| = 1.00 ΔV^+^) in the injected current range (±600, ± 500, ± 400, and ± 300 pA; STAR Methods). (E), long-pulse voltage recordings at the soma in blocker conditions from the same cells as in D. (F), plot of the stepwise linearity of all somatic long-pulse recordings (|ΔV^–^| = 0.912 ΔV^+^, n = 19 cells) in the injected current range (±120, ± 100, ± 80, ± 60, ± 40, and ± 30 pA). (G), Two example axonal recording traces from axo-somatic short-pulse injections (2 ms, ± 600 pA) in blocker conditions from the minimum to maximum recording distances (~140–830 μm, n = 6 cells). (H), stepwise linearity plot of the axonal responses (|ΔV^–^| = 0.994 ΔV^+^) evoked by somatic current injections (±600, ± 500, ± 400, and ± 300 pA).

**Figure S2:**
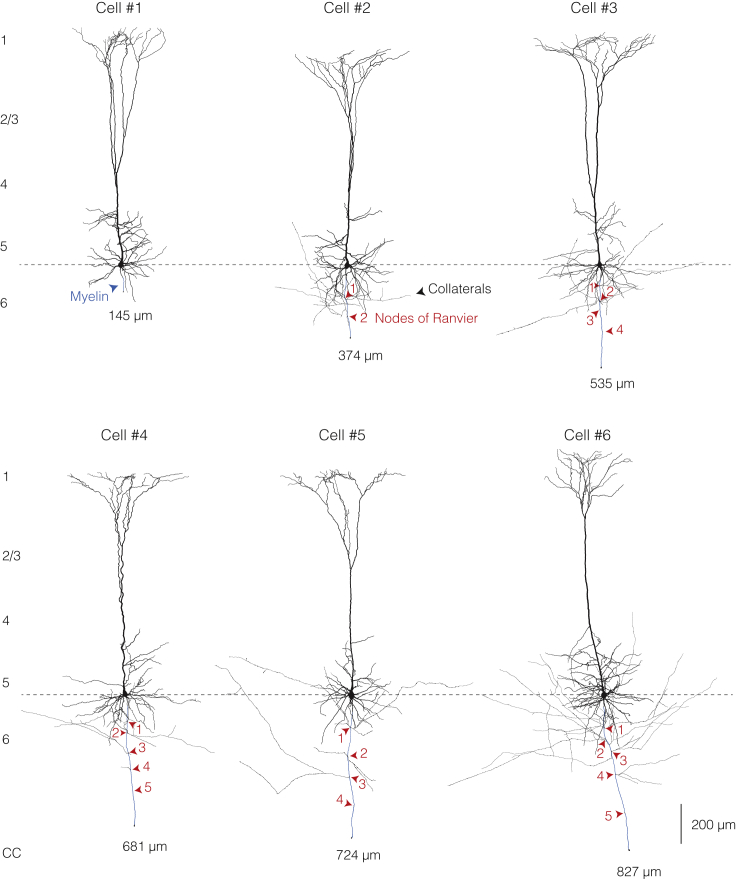
Full Morphological Reconstructions of Neurons Used for Modeling, Related to Figure 1 Reconstructed thick-tufted L5 pyramidal neurons from the primary somatosensory cortex of the rat recorded with axo-somatic dual whole-cell recording. Cell numbers refer to the model numbers elsewhere in the text. Red arrows indicate the location of the identified nodes of Ranvier. Regions of myelinated internodes are shown in blue.

**Figure S3:**
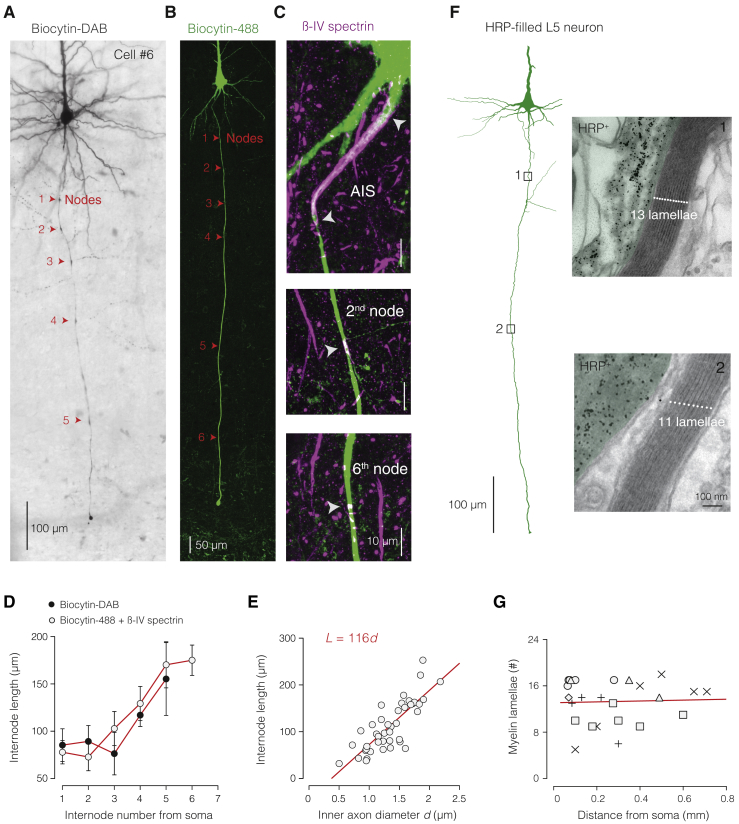
Nodal and Internodal Architecture of Thick-Tufted L5 Axons, Related to Figure 1 (**A**), Low magnification photomicrograph of a biocytin-stained cell (cell #6). Nodes were identified by an increased staining intensity and/or branch point, indicated by the position of arrows (red). (**B**), Immuofluorescence staining for biocytin-streptavidin (green) and the nodal/AIS marker βIV spectrin (magenta). Nodes of Ranvier, identified by the overlap of spectrin and biocytin, are indicated with arrows (red). (**C**), High magnification of the AIS and two nodes (2^nd^ and 6^th^) from the axon shown in (**B**). (**D**), Comparison of internode length as a function of internode number for the biocytin-stained axons (cells #2–6) and the immunofluorescence identified L5 axons (n = 8) revealed a similarity in the sequence of internode lengths (two-way ANOVA, group-internode interaction (p > 0.890). The first 2–3 internodes are short, with collaterals emerging from the nodes of Ranvier. With increasing distance from the soma, internodes are progressively longer and lack collaterals. Data represent mean ± SEM (**E**), Internode length (*L*) scaled linearly with axon core diameter *d*, based on all immunofluorescence-identified internodes, (n = 42; *r*^2^ = 0.653). (**F**), Example of a L5 neuron filled with HRP and recovered for EM analysis. EM images are shown for the two indicated locations. (**G**), The number of myelin lamellae was constant with distance from the soma (red line, linear regression slope ≈0, *F* test p > 0.836; *R*^2^ = 0.00191, n = 25 internodes from 6 axons, represented by different symbols).

**Figure S4:**
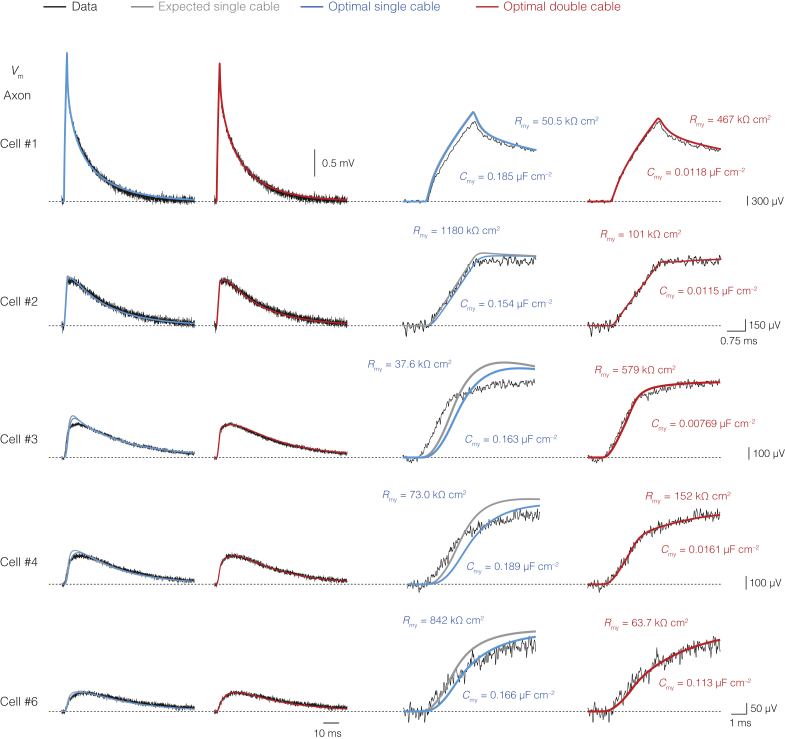
Single and Double-Cable Optimizations for the Axonal Voltage Responses, Related to Figure 1 Left, voltage response of recorded axonal voltage transients from cells #1–4 and #6 fit by either the minimum expected SC model (gray; *R*_my_ = 20 × *R*_m_, *C*_my_ = 0.05 × *C*_m_), the optimal SC (blue) or the DC model (red). Right, higher magnification of the first milliseconds of the fit. Corresponding values for myelin resistance and capacitance are indicated. Note the improved fits for all models with DC circuits implemented at the internodes (red) in comparison to expected (gray) and optimized SC circuits (blue). Neuron morphologies shown in Figure S2.

Using two patch-clamp electrodes, we simultaneously recorded *V*_m_ at the soma and axon up to ∼830 μm away from the soma in rat thick-tufted layer 5 (L5) pyramidal neurons (Kole and Popovic, 2016, Kole et al., 2007) (Figure 1A). To solve the combined passive cable parameters for the neuron and its myelin sheath, capturing transient cable elements such as axial resistance and capacitance, we injected brief current pulses at the soma and recorded passive voltage responses (>300) averaged each for the soma and axon end (STAR Methods; Figures 1 and S1). From six high-quality recordings, we developed anatomically detailed cable models (Figures 1 and S2). The total length of the primary axon ranged from 145 to 830 μm, encompassing 1–6 myelinated internodes, increasing in length with distance from the soma (Figure S3). Within biophysical bounds (Table S1), we began by optimizing the myelinated axon as an SC (Figure 1B). Optimization was performed simultaneously for neuronal intracellular resistivity (*R*_i_), specific membrane resistance (*R*_m_), and capacitance (*C*_m_), as well as specific myelin sheath resistance (*R*_my_) and capacitance (*C*_my_), taking advantage of an optimization algorithm we developed for unsupervised solution selection (Figure 1B; STAR Methods). The optimal SC solution predicted high *R*_my_ but only modestly low *C*_my_ values and could not capture the rapid rising phase in axonal *V*_m_ in combination with amplitude attenuation, robustly observed in all axonal recordings (Figures 1B and S4; Table S2).

To examine whether parameters were accurately estimated with soma-axon voltage transients only, we made additional simultaneous recordings from somatic, dendritic, and axon initial segment sites in L5 neurons but found no difference between *R*_i_, *R*_m_ and *C*_m_, thus suggesting the main neuronal parameters were well constrained (Tables S2 and S3; p > 0.255–0.999, STAR Methods; n = 12 cells). Next, in a bid to improve the SC fit error and better understand the dynamics of the axonal response, we kept the optimal solutions for *R*_i_, *R*_m_, and *C*_m_ and fixed the myelin parameters *R*_my_ and *C*_my_ to their expected values, based on 10 myelin lamellae (2 myelin membranes per lamella). With the serial compaction of each myelin layer, *R*_my_ and *C*_my_ were accordingly fixed to 20 × *R*_m_ and 0.05 × *C*_m_, respectively (Equations 7, 8, 9, and 10; STAR Methods). The results showed that the expected SC captured the onset of the rising phase but not its second attenuated component, leading to an even larger fit error (Figures 1B, 1D, and S4).

While the experimentally observed sharp rise time in the axon (Figures 1 and S4) could reflect the smaller capacitive load of myelinated internodes, the presence of a second component suggests an additional resistive path. We therefore hypothesized that the submyelin space may be conducting and connected to ground at the paranodes, forming a DC. To implement submyelin conduction, we added an axial resistor (*r*_pa_) longitudinal to the entire length of the reconstructed internode and an axial paranodal resistance parameter at its edges (*r*_pn_, fixed length of 2.3 μm) through which current leaves to ground (Figure 1C; Table S1; STAR Methods). Subsequently, we re-optimized the models with *r*_pa_, *r*_pn_, and the neuronal and myelin membrane parameters (*R*_i_, *R*_m_, *C*_m_, and *R*_my_, *C*_my_, respectively). The optimized DC model results demonstrated a significantly improved fit of the biphasic axonal transients, as well as the somatic transient across all six models (Figures 1C, 1D, and S4; Table S4). Moreover, even without *r*_pn_, the DC model produced a better fit and improved combined axo-somatic error relative to those of the expected and optimal SC models (p < 0.0001; STAR Methods). Finally, to assess the validity of the passive cable solutions and control for the number of parameters, different sources of error were explored via uncertainty analyses, SC and DC circuits were compared via sensitivity analyses, and alternative SC circuits were optimized with separate myelin sheath resistance and capacitance parameters for each internode (1–6 internodes), together for over 2 million core hours on two supercomputers at the Neuroscience Gateway (Sivagnanam et al., 2013; STAR Methods). The results of these analyses indicated that the combined axo-somatic fit error was consistently larger than DC model solutions (p < 0.0001; STAR Methods).

Taken together, our experimentally constrained passive cable modeling reveals a conducting submyelin axial pathway as integral to the equivalent circuit of a myelinated internode.

### DC Parameters Predict the Myelin Ultrastructure

**Figure 2:**
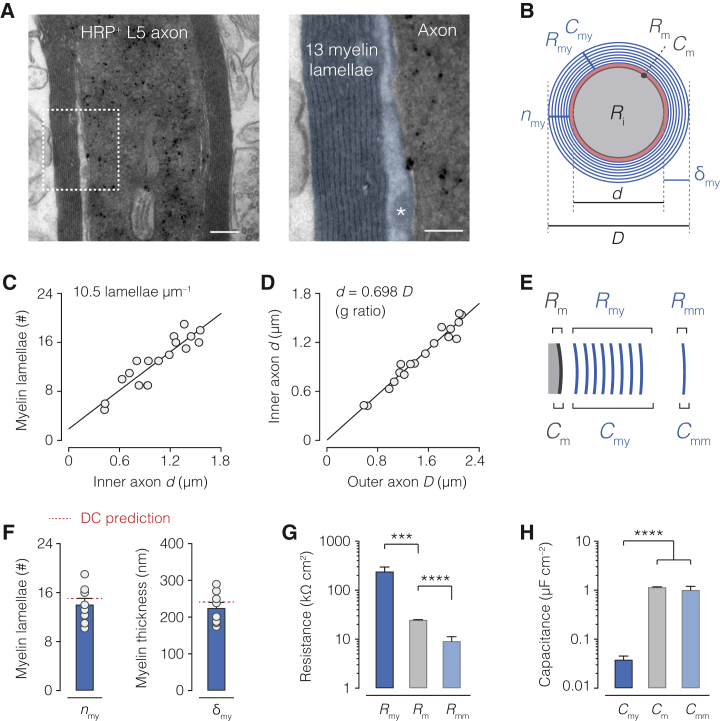
Myelin Sheath Ultrastructure Is Consistent with Double-Cable Parameters (A) Left, EM image of a L5 axon labeled with HRP (black precipitation). Right, higher magnification (white dotted box in left) revealing myelin membranes and lamellae (here, *n*_my_ = 13), false colored in blue. The cytoplasmic loop is marked with an asterisk. Scale bars, 200 nm (left) and 100 nm (right). (B) Cross-sectional schematic showing internode parameters with their radial circuit correlates, including those for myelin (blue: *R*_my_, *C*_my_, *n*_my_, and δ_my_) and axon core (gray: *R*_m_, *C*_m_, and *R*_i_). Also shown are axon core diameter (*d*) and total fiber diameter (*D*). (C) Linear variation of myelin lamellae with internodal *d* (linear regression, 10.5 lamella μm^–1^, *F* test p < 0.0001, *R*^2^ = 0.823; n = 18 internodes from 8 axons). (D) Linear variation of inner axon *d* with outer axon *D* reveals a *g* ratio of 0.698 (linear regression, *F* test p < 0.0001, *R*^2^ = 0.959; n = 18 internodes from 8 axons). (E) Schematic illustration of *R*_my_ and *C*_my_ composed by each myelin membrane (*R*_mm_ and *C*_mm_). (F) Left and right, EM estimates for *n*_my_ and δ_my_, respectively. Data are shown as mean ± SEM and individual internodes (open circles, n = 8). Red dotted line, DC model prediction (n = 6 axons). (G) Population data for radial resistance values in DC models (Friedman test with Dunn’s correction p < 0.001 *R*_my_ versus *R*_m_; p < 0.0001 *R*_m_ versus *R*_mm_; n = 6 models). (See Equations 7 and 8; STAR Methods.) (H) Population data for DC model estimates (Friedman test with Dunn’s correction p < 0.0001 *C*_my_ versus *C*_m_ and *C*_mm_; p = 0.0429 *C*_m_ versus *C*_mm_; n = 6 models). Note, both *C*_mm_ and *C*_m_ are near 1 μF cm^–2^ (Equations 9 and 10; STAR Methods). Data represent mean ± SEM.

To test whether the cable properties predict features of the myelin ultrastructure, we examined biocytin- or horseradish peroxidase (HRP)-filled thick-tufted L5 neurons, including 4 of the 6 simulated neurons post hoc with EM (Figures 2A and 2B). The observed number of myelin lamellae (*n*_my_) varied between axons (range: 5–19 lamellae, Figure 2C) but was constant with distance from the soma on average (Figure S3). The *n*_my_ scaled linearly with inner axon diameter, resulting in a ratio of inner to outer axon diameter, or *g* ratio, of 0.698 (n = 18 internodes from 9 axons, Figure 2D), as expected for central nervous system axons (Waxman and Swadlow, 1977).

We next examined whether the optimized SC or DC cable parameters could predict *n*_my_ and the thickness of the myelin sheath (δ_my_), as well as the resistance and capacitance of each myelin membrane (*R*_mm_ and *C*_mm_, respectively; Figures 2B and 2E; Equations 7, 8, 9, 10, 11, and 12). Given the serial compaction of each myelin layer, total myelin sheath resistance could be estimated as the sum of the resistance of each layer, and the capacitance the reciprocal of the reciprocal sum of the capacitance of each layer. Therefore, *n*_my_ could be directly predicted from total myelin sheath capacitance (*C*_my_), by dividing each model *C*_m_ by twice *C*_my_. The DC parameters predicted an average of ∼15 myelin lamellae, the same as observed in EM (∼14 lamellae, Mann-Whitney p = 0.345; Figure 2F). Furthermore, since the thickness of the myelin sheath separates two parallel capacitor plates, δ_my_ may be predicted by deriving *C*_my_ as the sum of the separation of each myelin membrane capacitor. This relationship predicted an average δ_my_ of 242 nm, well within range of the measured δ_my_ of 225 nm (Figure 2F). In contrast, the SC parameters predicted a substantially thinner myelin sheath of only ∼4 lamellae (predicted δ_my_ = 29.5 nm and *n*_my_ = 3.68, respectively; Table S2), yielding poorer predictions compared to the DC parameters (Kruskal-Wallis with Dunn’s correction, SC versus EM p = 0.00970, DC versus EM p = 0.930).

Since myelin sheath resistance and capacitance is composed by a series of connected myelin membrane resistances and capacitances, we could estimate the specific properties of individual myelin membranes by using the average number of myelin membranes, accordingly dividing *R*_my_ to obtain *R*_mm_ or multiplying with *C*_my_ to predict *C*_mm_ (Figure 2G). Interestingly, although the insulation provided by the total myelin sheath resistance was an order of magnitude greater than that of the axolemma, the resistance of a single myelin membrane was more than twice lower (*R*_mm_ = 8.56 ± 3.27 kΩ cm^2^; range: 2.28–20.6, n = 6; Figure 2H). A low specific myelin membrane resistance is in good agreement with values obtained from direct recordings of oligodendrocyte precursor cells (4.1 kΩ cm^2^; Chan et al., 2013) and numerical predictions of corpus callosum myelin membranes (1.8 up to 16 kΩ cm^2^ per membrane; Bakiri et al., 2011). Finally, the specific membrane capacitance for each myelin membrane was estimated at 1 μF cm^–2^ (1.00 ± 0.199 μF cm^–2^, n = 6; Figure 2H), close to the biological constant for phospholipid bilayer membranes (Gentet et al., 2000).

Together, these results show that our experimentally solved DC cable parameters for the myelin sheath are internally consistent and predict the ultrastructure of the myelin sheath.

### Conductivity in the Submyelin Space

**Figure 3:**
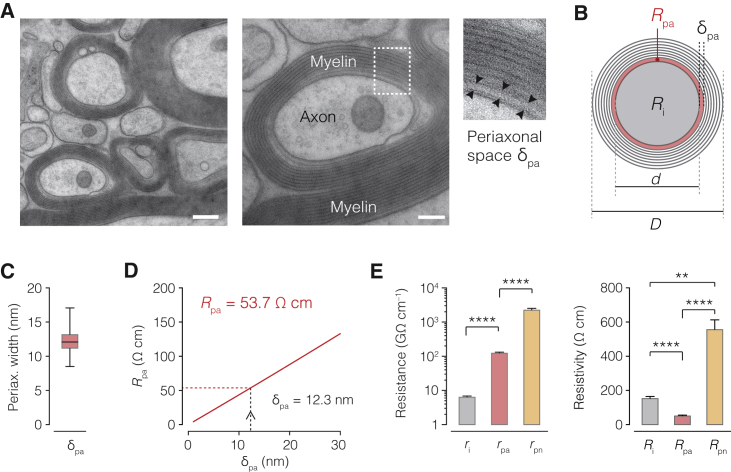
Ultrastructure of the Periaxonal Space Reveals a Low-Resistivity Pathway Relatively Sealed at Paranodes (A) Left, HPF EM image of the rat corpus callosum. Middle, higher magnification of one axon shown with the ROI (white dotted line). Right, 2.3 × magnification of the ROI. Periaxonal width (δ_pa_) between the outer axon and inner myelin (black arrowheads). Scale bars, left 200 nm and middle 100 nm. (B) Schematic of cross-sectional myelinated axon showing the axial circuit correlates for axon core (*R*_i_, black) and periaxonal space (*R*_pa_ and δ_pa_, red). *d* and *D* as in Figure 2. (C) Boxplot of δ_pa_ shows median and 25^th^ to 75^th^ percentiles and min-max values (black bars; n = 195 axons from 3 animals). (D) Plot of average periaxonal resistivity *R*_pa_ predicted from possible δ_pa_ values (red line, Equation 5). With δ_pa_ = 12.3 nm, *R*_pa_ is 53.7 Ω cm (dotted lines). (E) Left, comparison of optimized *r*_i_, *r*_pa_, and *r*_pn_. Right, corresponding *R*_i_, *R*_pa_, and *R*_pn_ based on δ_pa_ = 12.3 nm and δ_pn_ = 7.4 nm (Nans et al., 2011) (Table S4 and Equations 4 and 5; Friedman test with Dunn’s correction, ****p < 0.0001 and **p < 0.01; n = 6 neurons). Data are represented as mean ± SEM.

To determine how axial resistance corresponds to the submyelin space and how easily charges can flow therein, we calculated the axial resistivity. To do so, we converted the solved axial resistance in the periaxonal and paranodal spaces (∼125 GΩ cm^–1^ and ∼2.45 TΩ cm^–1^, respectively; Table S4) into resistivity (units of Ω cm, see Equation 5) by multiplying with the corresponding cross-sectional areas. To obtain reliable quantitative estimates of periaxonal space width (δ_pa_), we used a high-pressure freeze-substitution EM approach (HPF EM; Möbius et al., 2016) and measured the intercellular distance between the outside borders of the adaxonal myelin membrane and internodal axolemma (Figure 3A). The results showed that δ_pa_ was on average 12.3 nm (±0.192, range: 8.50–17.1 nm; Figure 3B), in close agreement with previous estimates (Li et al., 1994, Montag et al., 1994) and the crystal structure dimensions of myelin-associated glycoprotein (MAG), an important transmembrane protein in the axon-myelin space (∼10 nm; Pronker et al., 2016). Using this average δ_pa_ value together with the biophysical relationship between axial resistance and cross sectional area, we could estimate the axial resistivity of the periaxonal space fluid (*R*_pa_; see Equation 5, Figure 3C). *R*_pa_ was predicted at 53.7 Ω cm, strikingly close to the measured resistivity of extracellular fluid in the mammalian cerebral cortex (∼55 Ω cm at 35°C) (Fenstermacher et al., 1970). Next, to estimate axial resistivity for the fluid path through the paranode (*R*_pn_), we used a previously experimentally determined value of 7.4 nm (Nans et al., 2011), corresponding to a *R*_pn_ value of ∼550 Ω cm (Equation 5, n = 6 axons; Figure 3E). *R*_pn_ is thus on average 10 × higher than *R*_pa_, consistent with the presence of transverse bands (Hirano and Dembitzer, 1969, Rosenbluth et al., 2013).

These empirical estimates of the submyelin ultrastructure and its axial resistivity indicate that the nanoscale (∼12 nm) periaxonal space is 3 × more conductive than the axon core, with submyelin conduction relatively restricted at the paranodal domains.

### Submyelin Resistance Produces Saltatory Conduction in the Temporal Domain

**Figure 4:**
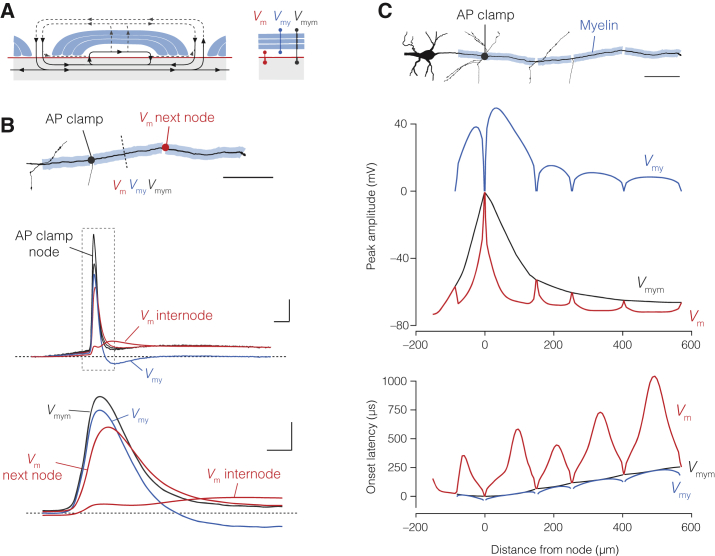
A Double-Cable Model Generates Amplitude and Temporal Saltation in *V*_m_ (A) Left, schematic of currents in a DC internode including the submyelin and extracellular current return pathways (dotted lines). Right, the three potentials in the DC model (transaxonal *V*_m_, between axon core and periaxonal space, transmyelin *V*_my_, between periaxonal and extracellular spaces, and transfiber *V*_mym_, between axon core and extracellular). (B) Top, part of the morphology of cell #5 indicating site for the “AP voltage clamp” at the 3^rd^ node. Active conductances are not included in this model. Middle, *V*_m_ (red), *V*_my_ (blue), and *V*_mym_ (black) shown for the middle of the following internode (red dotted line) and the resulting *V*_m_ at the next node (red). Note the afterhyperpolarization in *V*_my_ and depolarization transients in *V*_m_. Bottom, expanded view of internodal potentials as well as *V*_m_ at the next node. Scale bars, top, 100 μm. Middle, 20 mV and 1 ms. Bottom, 20 mV and 200 μs. (C) Top, part of cell #5 morphology with AP clamp applied to the 1^st^ node. Scale bar, 100 μm. Middle, spatial profiles of maximal *V*_m_, *V*_my_, and *V*_mym_. Bottom, spatial profile of the onset latencies of APs. Note the gradual amplitude and temporal saltation in *V*_m_.

How does submyelin conduction change AP propagation along the myelinated axon? To answer this question, we made model simulations. Following previous terminology (Halter and Clark, 1991), the three elemental voltages in a DC circuit are represented by, first, neuronal membrane *V*_m_, the potential difference between axon core and periaxonal space (also called transaxonal *V*_m_). The second by transmyelin *V*_my_, the potential difference between periaxonal and extracellular spaces. The third voltage element represents the total membrane potential *V*_mym_, between the axon core and extracellular space (also called transfiber *V*_mym_, Figure 4A).

To examine the role of submyelin conduction, the experimentally recorded axonal AP of cell #5 was used as a voltage-command potential (“AP clamp”) at the third node, propagating through its optimized DC internodes without active conductances (Figure 4B; Table S4; Figure S2). The model simulations demonstrated that *V*_m_ at downstream nodes depolarized *faster* and *earlier* than the transaxonal *V*_m_ of the upstream internodal axolemma (Figure 4B). In addition, the amplitude of the next node’s *V*_m_ was higher than that of the upstream internodal axolemma’s *V*_m_. The waveforms were also highly distinct during the second depolarizing peak in internodal *V*_m_, during which *V*_my_ showed a direction opposite to *V*_m_ and hyperpolarized. To explore the voltage profiles across the entire axon (∼725 μm), the AP clamp was applied to the first node (Figure 4C). The maximum amplitudes in *V*_m_ were higher in nodes and gradually attenuated within the internode, mirrored by opposite deflections in maximum *V*_my_. Furthermore, the spatial distribution of onset latencies in *V*_m_ revealed a gradual pattern of deceleration and acceleration repeated in each internode. Both the maximum onset delays and minimum amplitudes in *V*_m_ occur at around the middle of each internode, with *V*_m_ in longer internodes more attenuated and slower rising than *V*_m_ in shorter internodes. These results can be explained by the initial rapid charging of the upstream end of the myelin sheath generating a feedforward charging of the downstream end, forming the basis for the velocity and shape of the next node’s response.

Together, these results highlight how a conducting submyelin space produces gradually attenuating and slower internodal waves, with nodal potentials appearing repeatedly displaced back in time, creating the effect of what we here call *temporal saltation*.

### Voltage-Calibrated Optical Recording of Temporal Saltation at the Circuit Level

**Figure 5:**
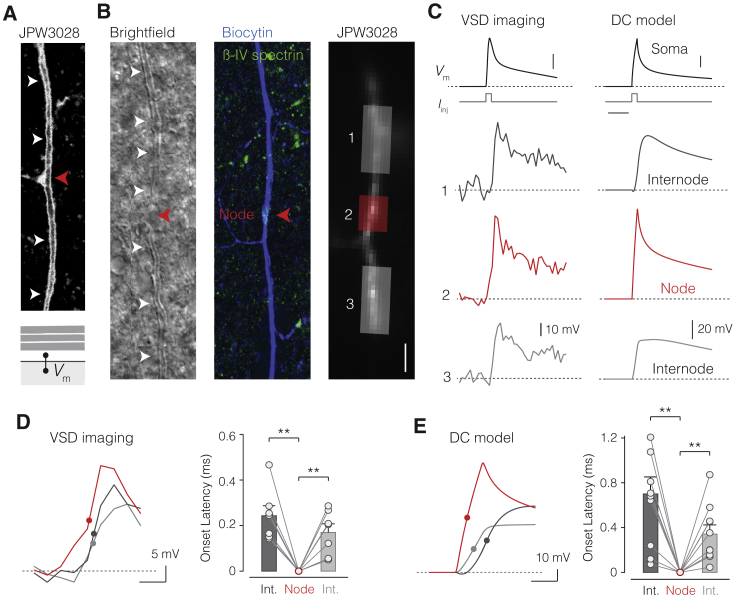
Optical Recording of Passive Transaxonal *V*_m_ Reveals Temporal Saltation (A) Top, confocal image of JPW3028 (VSD). Note, the dye remains in a single axolemma membrane (*V*_m_) at the node (red arrow) and internodes (white arrows). Bottom, schematic of internodal *V*_m_, between core and submyelin space. (B) Left, bright-field image of an axon with myelin (white arrows), putative node (red arrow). Middle, same axon stained with biocytin-streptavidin (blue) and βIV spectrin (green). Right, epifluorescence of VSD in the axon. Transparent areas correspond to the ROIs for node (red) and upstream (dark gray) and downstream internode (light gray). Scale bar, 10 μm. (C) Left, voltage-calibrated VSD fluorescence traces (2 kHz acquisition) in response to a brief current injection in the soma (1 ms, 50 nA; top). Right, corresponding DC model prediction for *V*_m_ (cell #3) at comparable locations (2 kHz simulation). Scale bars, top, 25 mV (VSD), 50 mV (model), and 4 ms. (D) Left, VSD traces overlaid and expanded in time. Dots, 50% onset time. Right, population data of onset latencies (one-way repeated-measures [RM] ANOVA with Bonferroni’s correction, node versus preceding internode (Int.) p < 0.0025 and node versus proceeding internode p < 0.0025; n = 7 axons). Scale bar, 1 ms. (E) Left, DC model prediction for *V*_m_. Right, population data (one-way RM ANOVA with Bonferroni’s correction, node versus preceding internode p < 0.0021 and node versus proceeding internode p < 0.0037; n = 10 node/internodes, from n = 5 axons). Columns and error bars represent mean ± SEM. Circles and connected lines represent individual first and second nodes of Ranvier paired with their adjacent internodes. Scale bar, 1 ms. See also Figure S5.

**Figure S5:**
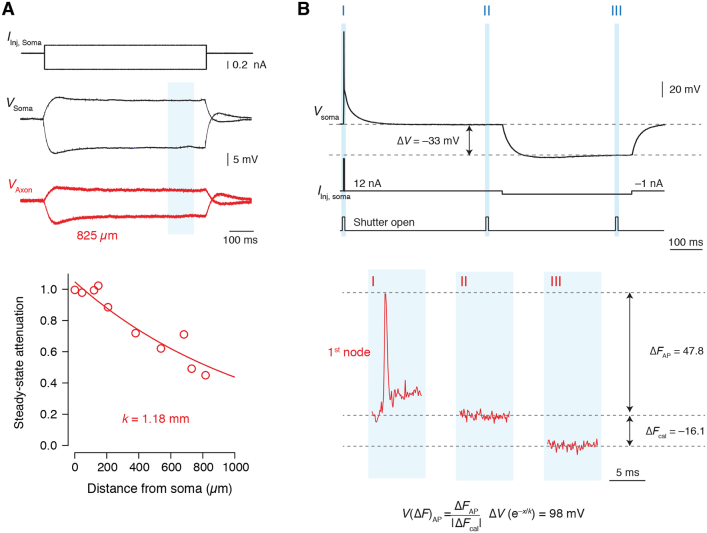
Voltage-Calibration Protocol for Optical Recordings along the Axon, Related to Figures 5 and 6 (**A**), Top, example traces of a simultaneous axo-somatic whole-cell current-clamp recording in normal extracellular solution. Subthreshold depolarizing and hyperpolarizing steady-state current injections at the soma evoked voltage responses at the soma (black) and axon (red) measured in the indicated region (blue). Bottom, averaged amplitudes were normalized to the somatic amplitude and plotted as a function of recording distance from the soma. Data were fit according to an exponential function (*V*(*x*) = *e*^–*x*/*k*^), with *k* being 1.18 mm (n = 10). (**B**), Top, protocol for voltage calibration. Within each imaging trial, a fast passive transient (Figure 5) or AP (Figure 6) or was evoked by current injections (1 ms, 50 nA or 3 ms, ~10 nA, respectively), followed by a long interval during which the *V*_m_ returned to baseline. Example traces show the protocol for an AP. Thereupon, a large hyperpolarizing current step was applied (up to –1.5 nA, 400 ms) to obtain a substantial steady-state potential. To reduce phototoxicity but optimally detect fluorescence during the hyperpolarizing pulse, total light exposure per trial was 21 ms divided into 3 × 7 ms segments (I-III, indicated in blue regions). Bottom, closer view of shutter segments and optically-recorded voltage responses. The first segment coincided with the fast transient or AP-generating current pulse, the second was immediately before the negative current injection, and the third was just prior to the end of the negative current injection near steady-state of the membrane potential. The difference in fluorescence between the baseline and plateau Δ*F*_cal_ was used to calibrate the depolarization (Figure 5) or AP fluorescence signal (Figure 6), corrected for the known steady-state axo-somatic voltage attenuation (based on *k*), yielding the VSD-calibrated *V*(Δ*F*)_AP_.

The spatial profile of onset latency resembles previous findings using VSD imaging of transaxonal *V*_m_ along the axon of L5 pyramidal neurons (Palmer and Stuart, 2006, Popovic et al., 2011). However, even without Na^+^ channels, the presence of a myelin sheath with a conducting submyelin space may alone suffice to temporally advance *V*_m_ at nodes relative to upstream and downstream internodes (Figure 4). To test and quantify this experimentally, we performed VSD imaging using the highly sensitive electrochromic transmembrane dye JPW3028, reporting *V*_m_ with a delay of ∼11 μs (Ehrenberg et al., 1987). The lipophilic styryl dye integrates into the neuronal plasma membrane enabling a direct optical readout of transaxonal *V*_m_ beneath the myelin sheath and at nodes (Figure 5A; Popovic et al., 2011). To obtain passive conditions, we used a solution with extracellular blockers (Figures 1 and S1; STAR Methods). Furthermore, we calibrated the fluorescence signals (ΔF/F) to absolute *V*_m_ changes (Figure S5). The location of nodes was identified by interruptions of the myelin sheath as imaged under oblique contrast. Post hoc staining further guided a micrometer-precise analysis of the regions of interest (ROIs; spatial resolution of 1 μm) to the corresponding nodes (first or second) and their upstream and downstream internodes (Figures 5B and 5C). The results indicated that optically recorded *V*_m_ in nodes was temporally advanced relative to the *V*_m_ transients in adjacent upstream and downstream internodes (Figures 5C and 5D). The onset latency (the time to 50% of the peak amplitude) revealed an advancement of ∼200 μs at the nodes of Ranvier with respect to internodal axolemma at ∼5 μm distance from the node (p < 0.01, Figures 5C and 5D, n = 7 axons). Comparing this experimental result with predictions from optimal DC models showed that *V*_m_ at the nodes was temporally advanced by an average of ∼400 μs relative to upstream or downstream internodal *V*_m_ ∼5 μm from the node (Figure 5E; n = 5 DC models with at least two nodes).

Together, these results establish temporal saltation in passive myelinated axons and delayed yet substantial internodal depolarizations, both consistent with the optimized models and a DC architecture.

### High-Speed Optical Recording of APs Reveals Spatial and Temporal Structure of Saltation

**Figure 6:**
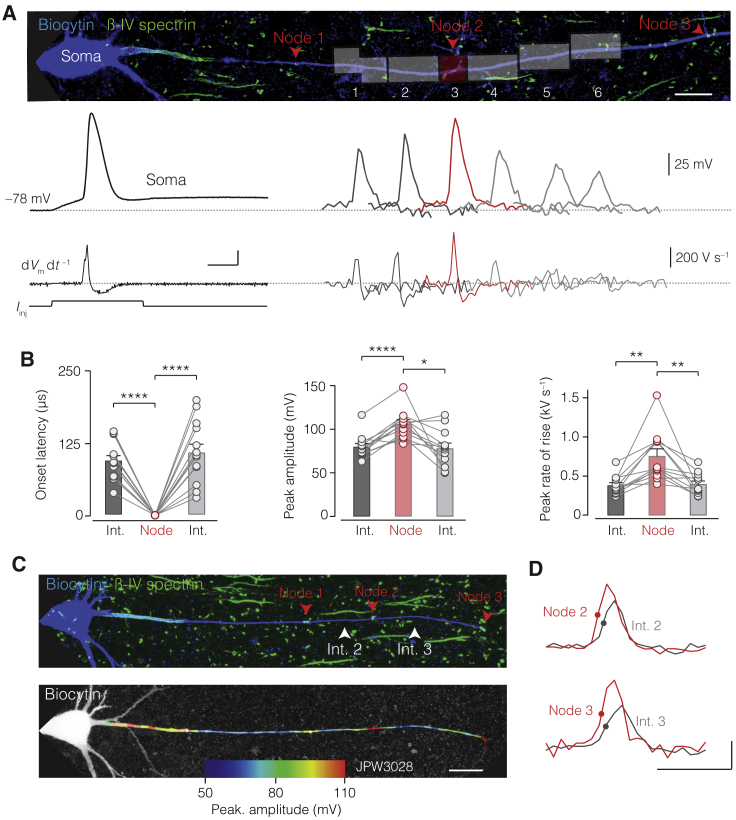
High-Speed Optical Recording of Transaxonal *V*_m_ Reveals Temporal and Amplitude Saltation (A) Top, *z*-projected confocal image of a biocytin-filled L5 axon (blue) with βIV spectrin (green, red arrows). Transparent areas (1–3 pixels), imaged regions of the node (red), upstream and downstream internode (dark and light gray, respectively). Bottom, voltage-calibrated optical recordings of *V*_m_ (20 kHz rate) and temporal derivatives (d*V*_m_ d*t*^–^^1^) from the indicated ROIs. Scale bars, top, 10 μm, bottom left, 400 V s^–1^ and 1 ms. (B) Population data reveal increased onset latency, lower peak amplitude, and reduced rate of rise relative to adjacent nodes. Bars indicate mean ± SEM, and circles and connected lines indicate individual axons (one-way RM ANOVA with Bonferroni correction. Latency, node versus preceding internode (int.) p < 0.0001 and proceeding int. p < 0.0001; amplitude: node versus preceding p < 0.0001 and proceeding p < 0.021; rate of rise, preceding p < 0.0032 and proceeding p < 0.01; n = 12 axons). (C) Top, *z*-projected confocal image of L5 axon. Bottom, axon overlaid with color-coded profile of the maximum *V*_m_ (10 kHz). Scale bar, 25 μm. (D) Example traces of voltage-calibrated APs. Note the saltation in time and amplitude. Scale bars, 50 mV, 1 ms. See also Figure S5 and Video S1.

A related prediction of the DC model is that during AP propagation, *V*_m_ along the internode is non-uniform, slower in its rise time and gradually attenuating in its peak amplitude (Figure 4). To test this experimentally, we performed VSD imaging of APs under physiological conditions, using the maximum temporal resolution of the camera (20 kHz) and taking advantage of our voltage calibration approach for fluorescence signals (Figure S5). Nodal APs across the axolemma (*V*_m_) were temporally preceding those at adjacent internodes by ∼100 μs (n = 12 nodes from 7 axons, Figures 6A and 6B). On average, the internodal APs were ∼25 mV smaller in amplitude and rose 3 × slower than at adjacent nodal axolemmal APs (d*V*_m_/dt internodes, ∼300 V s^–1^ versus ∼900 V s^–1^ in nodes, n = 12; Figure 6B). Furthermore, to explore the spatiotemporal pattern of APs along multiple nodes and the entire length of internodes, we imaged at a lower frame rate (10 kHz), enabling a view of the first ∼250 μm of the axon (Figure 6C). The recording showed that the pattern of saltatory propagation was reliably repeated along the first three internodes, showing both slower rise times and attenuated peak depolarizations while still reaching approximately –30 mV in the middle of the internode (Figures 6C and 6D; Video S1).

Video S1. Optically Recorded Saltatory Propagation of the Action Potential, Related to Figure 6 Top, transaxonal *V*_m_ experimentally recorded with voltage-calibrated VSD signals during AP initiation and saltatory propagation across the first three internodes. Color-coded membrane potentials are overlaid with the confocal *z*-stack of the same cell. Color scale shown bottom left. Bottom panel, the temporal evolution of optically recorded *V*_m_ plotted as spatial profile along the main axon against distance from the soma. The temporal resolution during fluorescence acquisition was 10 kHz but splined to 50 kHz for visualization purposes. The movie shows 3 ms of experimental recording at a 3500× reduction in speed.

These results demonstrate that saltatory conduction of *V*_m_ occurs both in the spatial and temporal domains, consistent with simulation predictions predicated on a DC circuit in the myelinated axon.

### A Conductance-Based DC Model Constrained to Voltage Recordings Reproduces the Structure of Saltation

**Figure 7:**
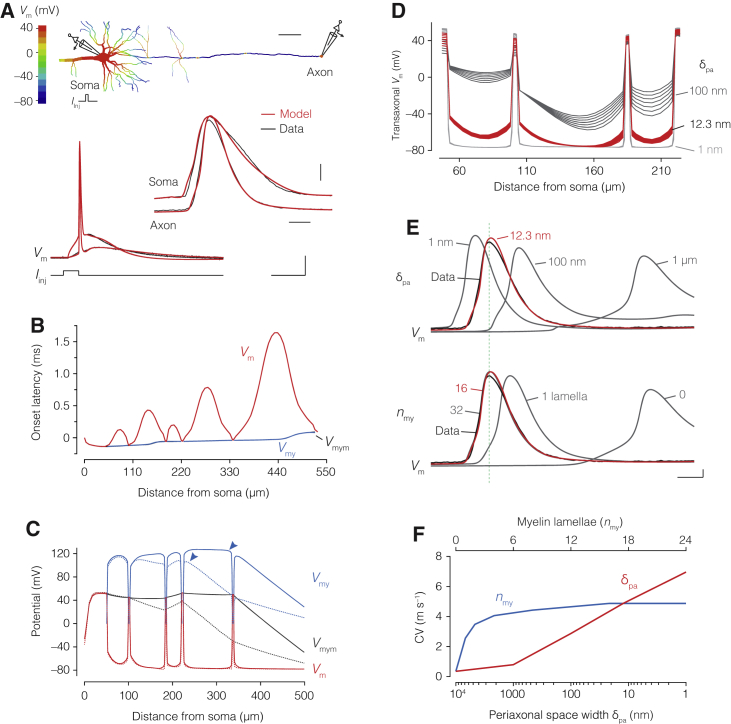
Action-Potential-Constrained Double-Cable Model Reproduces Saltatory Conduction (A) Top, color-coded spatial spread of *V*_m_ in cell #3. Bottom, overlay of experimentally recorded (black) and DC optimized APs from axon and soma (red). Scale bars, top to bottom, 50 μm, 20 mV, 250 μs, 20 mV, 10 ms. (B) Onset latency of the AP for *V*_m_ (red), *V*_my_ (blue), and *V*_mym_ (black), revealing temporal saltation of *V*_m_. (C) Spatial profile of *V*_m_, *V*_my_, and *V*_mym_ before and after the jump from node 3 (dotted line) to node 4 (continuous lines), illustrating the charge transfer via *V*_my_ (blue arrows, upstream and downstream ends). (D) Expanded view of the first 3 nodes and internodes showing *V*_m_ at 8 sequential time points during the AP peak in node 3 (80 μs) for optimal δ_pa_ (12.3 nm, red) in comparison to 1 and 100 nm (gray; Equation 5). Note the lack of depolarization within internodes for δ_pa_ = 1 nm. (E) Top and bottom, impact of δ_pa_ and *n*_my_ on CV. Optimal *n*_my_ was fixed to 16 (red), corresponding to the EM data. *n*_my_ variation was simulated by changing *R*_my_ and *C*_my_ (Equations 8 and 10). Traces temporally aligned to the soma AP (vertical line). Scale bars, 10 mV, 1 ms. (F) CV plotted as a function of δ_pa_ (red) and *n*_my_ (blue). See also Figure S6 and Videos S2, S3, and S4.

**Figure S6:**
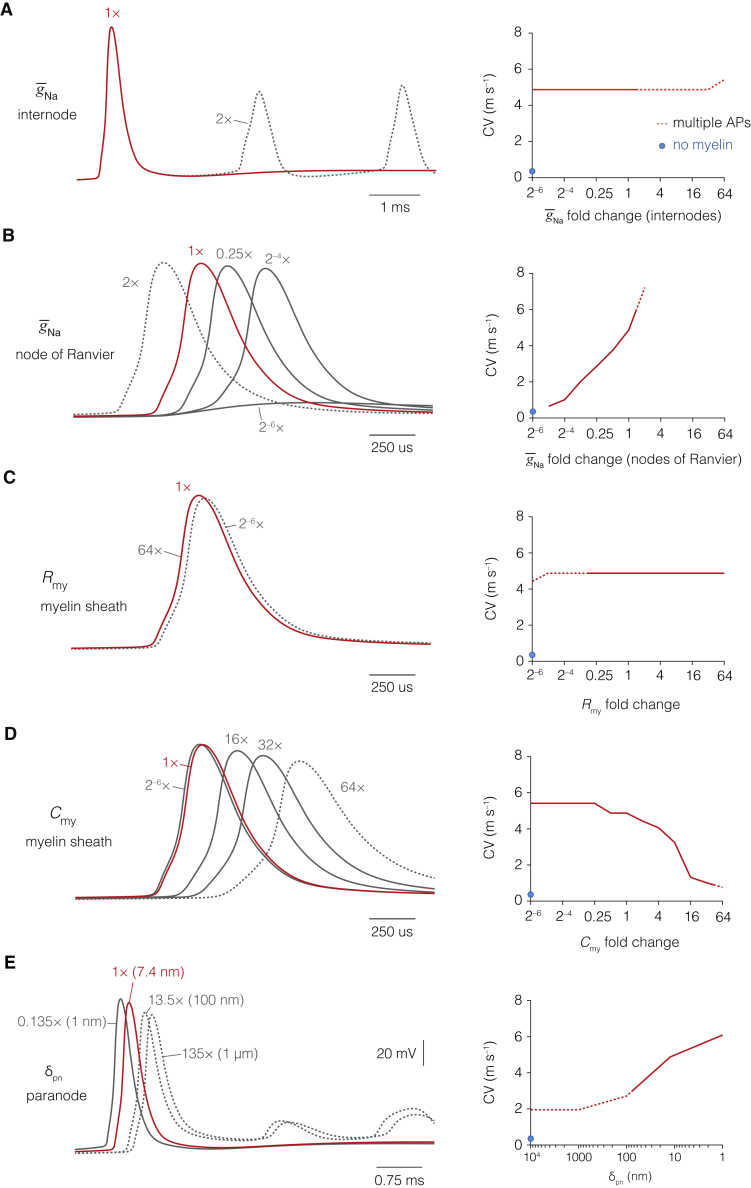
AP Conduction Velocity Depends upon Sodium Conductance at Nodes of Ranvier, Myelin Capacitance, and Submyelin Resistance, Related to Figure 7 (**A**), Conduction velocity (CV) was insensitive to changes in the peak sodium conductance density $\left({\bar{g}}_{Na}\right)$ at the internodal axolemma. However, increasing internodal ${\bar{g}}_{Na}$raised the probability of axonal spike generation (dotted line). Optimized trace is shown in red. (**B**), reducing ${\bar{g}}_{Na}$ in nodes of Ranvier below the optimized model (red) strongly decelerated AP propagation. Increasing nodal ${\bar{g}}_{Na}$ led to multiple APs as well as much faster CVs (dotted lines). (**C**), CV is independent of myelin sheath (*R*_my_) insulation but reducing *R*_my_ below 0.25 times its model value (red) led to an increase spiking activity (dotted lines). (**D**), increasing myelin *C*_my_ robustly decelerated AP velocity but little to no change was observed upon decreasing *C*_my_, suggesting *C*_my_ was optimized for a high CV. Optimized model is shown in red. Raising *C*_my_ extremely by > 64× led to multiple APs (dotted lines). (**E**), increasing δ_pn_ from 1 nm to 1 μm (with constant δ_pa_) decreased CV by approximately half. Optimized trace is shown in red. An increase in δ_pn_ of 10× and beyond increased excitability (dotted lines).

To explore how the periaxonal space impacts saltatory conduction, we used the optimized DC models of cells #3 and #6, representing the upper and lower estimated range for submyelin axial resistance, respectively (Table S4). The models were extended with active conductances, distributed along the dendrites, soma, axon initial segment, nodes, internodes and axon collaterals. They were initialized with the expected values, followed by manual and automated optimization constraining the model to the somatic and axonal APs. The results on the basis of the DC parameters showed an excellent fit, including the narrower shape of the axonal AP, its biphasic rising phase, afterdepolarization, and conduction velocity (CV; Figures 7A and S6; STAR Methods), obtained with low Na^+^ peak conductance density $\left({\bar{g}}_{Na}\right)$ in the internodes (<50 pS μm^–2^) and a much higher density in the nodes (<30 nS μm^–2^). Changing these showed that CV was strongly dependent on nodal ${\bar{g}}_{Na}$but essentially independent of internodal ${\bar{g}}_{Na}$, while the latter caused regenerative spiking when raised only 2-fold (Figure S6). Importantly, DC models robustly generated saltation of transaxonal *V*_m_ both in the spatial and temporal domains (Figures 7B and 7C; Videos S2 and S3). Plotting the three potentials revealed that, during the AP jump from the 3^rd^ to the 4^th^ node, primarily the myelin sheath capacitor was rapidly charged (Figure 7C). The upstream internodal region transfers this depolarization in a feedforward manner to the downstream end via the periaxonal space, exiting gradually to ground through the paranode. This pattern repeats itself at each internode, generating the jumping of an AP from node to node (Figure 7C; Video S4).

Video S2. Saltatory Propagation of the AP in a Double-Cable Model Simulation of Cell #3, Related to Figure 7 Spatial spread and temporal evolution of neuronal and transaxonal *V*_m_ during AP initiation and saltatory propagating in the optimized double cable model of cell #3. Top plot shows the spread of *V*_m_ along the reconstructed neuronal morphology. Membrane potential color-coded for each section with the scale shown bottom left. Bottom, temporal evolution of the *V*_m_ profile along soma and primary axon plotted against distance from the soma. Temporal resolution of the model was 100 kHz and the movie runs at 60 fps.

Video S3. Saltatory Propagation of the AP in a Double-Cable Model Simulation of Cell #6, Related to Figure 7 Top, spatial spread and temporal evolution of neuronal and transaxonal *V*_m_ during initiation and saltatory propagating of a single AP of the optimized double cable model cell #6. Membrane potential color-coded for each section with the scale shown bottom left. Bottom, temporal evolution of the *V*_m_ profile along soma and primary axon plotted against distance from the soma. Note the more gradual voltage drop and amplification in transaxonal *V*_m_ within the internode in comparison to cell #3 (Video S2). Temporal resolution of the model was 100 kHz and the movie runs at 60 fps.

Video S4. Spatiotemporal Spread of the Three Potentials in a Double-Cable Model Simulation of Cell #6, Related to Figure 7 Top, Spatial spread and temporal evolution of transaxonal *V*_m_ during initiation and saltatory propagating of a single AP of the optimized double cable model cell #6. Membrane potential color-coded for each section with the scale shown bottom left. Bottom, temporal evolution of the transaxonal *V*_m_ (red), transmyelin *V*_my_ (blue) and transfiber *V*_mym_ (dotted white) profiles along soma and primary axon plotted against distance from the soma. Temporal resolution of the model was 100 kHz and the movie runs at 60 fps.

Simulating the tightening of the periaxonal space to 1 nm (Equation 5; STAR Methods), while maintaining the same paranodal seal resistivity, produced a more rapid AP propagation associated with a binary internodal Δ*V*_m_ attenuating to nearly zero values within a few micrometers into the internode (Figures 7D and 7E). This binary mode of propagation is inconsistent with the gradual spread of *V*_m_ within the internode, as captured by optical recordings and optimized models (Figure 6; Videos S1, S2, and S3). Vice versa, widening the periaxonal space increased transaxonal depolarizations while substantially decelerating CV, with the velocity at a δ_pa_ of 1 μm reaching the same slow values as models without myelin (Figures 7E and 7F), consistent with previous models (Richardson et al., 2000, Young et al., 2013). Finally, to dissect the contribution of myelin to CV, the thickness of the myelin sheath in model cell #3 was varied from 1 to 32 lamellae (*n*_my_, Equations 7, 8, 9, and 10; STAR Methods). The results showed that CV was highly accelerated by only a few lamellae and fully optimized at the experimentally determined ∼16 lamellae, with modest gains beyond (Figures 7D and 7E). This acceleration of CV by addition of myelin membranes was mainly due to their capacitance-lowering effect rather than radial insulation (Figures 7E and S6).

Together, these results indicate that the conducting nanoscale periaxonal space, partially sealed at the paranodes, combined with a low-capacitance myelin sheath, are fundamental components of saltatory conduction.

## Discussion

We show independent lines of evidence for an anatomical and electrical separation of the axon and its myelin sheath by the submyelin space. The emergent DC properties were consistent with the low capacitance produced by ∼15 myelin lamellae, a conductive nanoscale periaxonal space (∼50 Ω cm) and electrical sealing of the paranodes (∼550 Ω cm; Figures 2 and 3). These findings are in agreement with earlier predicted incomplete insulation by the myelin sheath on the basis of experimental recordings (Barrett and Barrett, 1982, Blight and Someya, 1985, Funch and Faber, 1984) and provide a quantitative framework for axoplasmic, periaxonal, and paranodal resistivity, substantially advancing our understanding of the parameters to model submyelin conduction (Arancibia-Cárcamo et al., 2017, Blight, 1985, Dimitrov, 2005, Gow and Devaux, 2008, Halter and Clark, 1991, McIntyre et al., 2002, Richardson et al., 2000, Stephanova and Bostock, 1995, Young et al., 2013).

The spatial and temporal evolution of the transaxonal potentials beneath myelin is strikingly complex with gradually attenuating waves toward the middle of the internode (Figures 5 and 6). Such a pattern of AP propagation is incompatible with either a tightly sealed DC or SC circuit model of the internode (Huxley and Stämpfli, 1949, Tasaki, 1939), which would rather produce a binary propagation profile (Figure 7) and supports the concept that internodes are equivalent to weakly sealed coupled capacitors of the internodal axolemma and myelin sheath. In internodes with a submyelin nanocircuit, nodal APs start earlier at downstream nodes compared to upstream internodes, creating the phenomenon of nodal APs jumping across not only internodal space but also *time*, propagating as temporally advanced transaxonal waves before the slower upstream transaxonal potentials of the internode (Figures 6 and 7; Videos S1, S2, S3, and S4). These findings provide an explanation for the optically recorded pattern of onset latencies along the axon (Palmer and Stuart, 2006, Popovic et al., 2011) and the loss of temporal saltation in demyelinated axons (Hamada et al., 2017).

### Computational Modeling of Nanoscale Axo-Myelin Domains

The biophysical basis of the propagation pattern and local circuits in a DC internode are, however, complex and require further study. In a DC circuit alone, the various radial and axial interactions generate differential spatial and temporal voltage gradients between the myelin sheath and axolemma and are influenced by multiple factors including internode length or resistivity of both periaxonal and paranodal spaces. For example, transmyelin potentials not only rapidly depolarize but also rapidly repolarize to drive local-circuit currents that impact the time course of internodal axolemmal potentials (Video S4). Furthermore, questions remain regarding other anatomical specializations within internodes. While our simulation of the submyelin space was represented by two serially connected resistor pathways connected to ground, which sufficed to reproduce our experimental findings, the outer loops of the spirally wrapped myelin membranes are characterized by multiple tight junctions and mesaxonal pathways (Gow and Devaux, 2008, Nave and Werner, 2014, Shroff et al., 2011, Snaidero et al., 2014), for which DC models with additional radial and axial circuits within the myelin sheath have been previously proposed (Gow and Devaux, 2008, Stephanova, 2001). Another intriguing observation is the presence of spirally and longitudinally arranged inner mesaxon loops continuing into the myelin sheath and even linking the two opposing juxtaparanodal domains (Altevogt et al., 2002, Rash et al., 2016). Whether these mesaxonal loops play an electrical role in saltatory conduction or in K^+^ ion buffering during repetitive firing is unknown. Although the present model lacked an explicitly simulated juxtaparanodal domain, the observed ∼80 mV depolarizations at these sites (Figure 6) are likely also shunted by the opening of voltage-gated K^+^ channels and predicts substantial millimolar changes in K^+^ concentration in the nanoscale periaxonal space. To study the spatiotemporal gradients of intra-internodal K^+^ concentrations, future models involving Poisson-Nernst-Planck equations need to be developed to directly resolve electrodiffusion in three dimensions (Dimitrov, 2005, Lopreore et al., 2008).

### A Double-Layer Architecture for Myelinated Axons

Comparative analysis shows that myelin likely evolved four times independently across widely separated taxa and with distinct axo-myelin arrangements, differing in their cellular origin and degree of compactness, although each form of myelin ensheathment surrounds a fluid-filled submyelin space (Castelfranco and Hartline, 2016, Hartline and Colman, 2007). Notably, the giant axon of the *Penaeus* shrimp, characterized by a highly distinct axon-myelin organization such as fenestrated nodes, also exhibits a wide submyelin space (∼100 μm), which is filled with a gel-like substance with an axial resistivity of ∼25 Ω cm, producing one of the fastest recorded conduction velocities across nervous systems, up to ∼210 m s^–1^ (Kusano, 1966, Xu and Terakawa, 1999).

In contrast, the presence of a periaxonal space in mammalian axons is speed limiting (Figure 7; Young et al., 2013). The submyelin space may reflect an evolutionary trade-off between maximizing CV and depolarization of the internodal axolemma, enabling the activation of voltage-gated channels or axoplasmic Ca^2+^-dependent signaling pathways. Indeed, electrophysiological and imaging studies show that the internodal axolema contains a wide variety of voltage-gated ion channels (David et al., 1993, Shrager, 1989, Zhang and David, 2016). Moreover, the periaxonal space also plays an important metabolic role by supplying energy substrates from the myelin sheath into the internodal axon core, including lactate and pyruvate (Fünfschilling et al., 2012, Nave, 2010, Nave and Werner, 2014). In this view, an outstanding fundamental question remains on the extent of the depolarization of the inner tongue in non-compact myelin during AP propagation. The volume of this cytoplasmic collar is variable along the internode and the adaxonal myelin membrane expresses N-methyl-d-aspartate (NMDA) and α-amino-3-hydroxy-5-methyl-4-isoxazolepropionic acid (AMPA) glutamate receptors, as well as inward-rectifying K^+^ channels including Kir4.1 (Micu et al., 2018, Saab et al., 2016, Schirmer et al., 2018, Snaidero et al., 2014). If the adaxonal membrane depolarizes during saltatory conduction, this could act as a coincidence signal opening NMDA/AMPA receptors or closing Kir4.1 channels and conveying local activity-dependent information at a single AP resolution.

Our present model for saltatory conduction may open additional avenues for investigating the role of nanoscale cellular architecture in neuro-glial interactions, as well as provide an electrical framework for studying activity-dependent myelin plasticity and examine how pathophysiological defects in the myelin sheath and submyelin spaces may cause the conduction impairments observed in demyelinating diseases (Calabrese et al., 2015, Trapp and Nave, 2008).

## STAR★Methods

### Key Resources Table

**Table:** 

REAGENT or RESOURCE	SOURCE	IDENTIFIER
**Antibodies**		

Rabbit polyclonal anti-βIV-spectrin	M. Rasband (BCM)	N/A
Alexa488 – Streptavidin (1:500)	Thermo-Fisher	Cat#: S32354; RRID: AB_2336881

**Chemicals, Peptides, and Recombinant Proteins**		

Tetraethylammonium chloride	Sigma-Aldrich	Cat#: T2265
4-aminopyridine	Sigma-Aldrich	Cat#: A78403
Biocytin	Sigma-Aldrich	Cat#: B4261
Cadmium chloride dehydrate	Sigma-Aldrich	Cat#: 21097
Sodium cacodylate trihydrate	Sigma-Aldrich	Cat#: C0250
Tetrodotoxin citrate (TTX)	Tocris	Cat#: 1069
ZD-7288	Tocris	Cat#: 1000
XE-991 dihydrochloride	Tocris	Cat#: 2000
CNQX disodium salt	Tocris	Cat#: 1045
JPW 1114 (analog of JPW 3028)	Thermo-Fisher	Cat#: D6923
Osmium tetroxide	Merck Millipore	Cat#: 124505
Potassium ferricyanide	Merck Millipore	Cat#: 104973
Tissue-Tek OCT compound	Sakura	Cat#: 4583

**Critical Commercial Assays**		

Vectastain Elite ABC horseradish peroxidase kit	Vector Laboratories	Cat#: PK6100; RRID:AB_2336819
DAB horseradish peroxidase substrate kit	Vector Laboratories	Cat#: SK4100; RRID:AB_2336382
Epoxy embedding kit	Sigma-Aldrich	Cat#: 45359

**Deposited Data**		

Example NEURON model	This paper	https://modeldb.yale.edu/260967

**Experimental Models: Organisms/Strains**		

Rat: Wistar/Hannover; strain: HsdCpb:WU	Harlan Laboratories	Cat#: 13508588; RRID: RGD_13508588

**Software and Algorithms**		

Axograph X (version 1.5.4)	Axograph Scientific	RRID: SCR_014284
Neurolucida (version 11)	MicroBrightField	RRID: SCR_001775
NEURON (versions 7.3 to 7.5)	(Hines and Carnevale, 2001)	RRID: SCR_005393
MATLAB (R2015b and R2016a)	MathWorks	RRID: SCR_001622
NeuroPlex (version 10.1.1)	RedShirt Imaging	RRID: SCR_016193
Fiji (ImageJ)	(Schindelin et al., 2012)	RRID: SCR_002285
GraphPad Prism (versions 6 and 7)	GraphPad	RRID: SCR_002798
SPSS (version 23)	IBM Analytics	RRID: SCR_002865
Framework for creating and optimizing models	This paper	https://github.com/Kolelab
Compiled NEURON with one extracellular layer (OS X)	This paper	https://github.com/Kolelab
Voltage recording noisy traces remover	This paper	https://github.com/Kolelab
Voltage recording threshold calculator	This paper	https://github.com/Kolelab

**Other**		

Vibratome	Leica	VT1200
Patch-clamp amplifier	Dagan Corporation	BVC-700A
ITC-18 AD/DA converter	HEKA Elektronik	895036
Borosilicate patch pipettes	Harvard Apparatus	30-0060
NeuroCCD-SMQ imaging system	RedShirtImaging	NCS01
DPSS continuous wave solid-state laser (500 mW)	CNI Lasersystems	MLL-FN-532
Motorized Filter Wheel	Thorlabs	FW212CNEB

### Lead Contact and Materials Availability

This study did not generate new unique reagents. Further information, access to raw data and requests for resources and models should be directed to and will be fulfilled by the Lead Contact, Maarten H. P. Kole (m.kole@nin.knaw.nl).

### Experimental Model and Subject Details

All experiments were carried out according to guidelines approved by the animal ethics committee (DEC) of the Royal Netherlands Academy of Arts and Sciences (KNAW) under the protocol number NIN 12.13 or in accordance with the German animal welfare law and local regulations for animal experiments. We used young-adult male Wistar/Hannover rats (P30–90, Harlan Laboratories, strain HsdCpb:WU) kept on a 12 hour light–dark cycle and housed in environmentally-enriched cages in groups of 2-4. Animals had no previous experimental exposure and weighed 100-350 g at the time of experiment. Brain slices were made 3 hours after onset of the light cycle.

### Method Details

#### Electrophysiology

Animals were deeply anaesthetized by 3% isoflurane inhalation, decapitated and 300 μm parasagittal slices containing the primary somatosensory cortex were cut with a Vibratome (1200S, Leica Microsystems) within ice-cold artificial cerebrospinal fluid (ACSF) of the following composition (in mM): 125 NaCl, 3 KCl, 25 glucose, 25 NaHCO_3_, 1.25 NaH_2_PO_4_, 1 CaCl_2_, 6 MgCl_2_, saturated with 95% O_2_ and 5% CO_2_ (pH 7.4). Following a recovery period at 35°C for 45 minutes slices were stored at room temperature in the cutting ACSF. Slices were transferred to an upright microscope (BX51WI, Olympus Nederland BV) equipped with oblique illumination optics (WI-OBCD) and visualized using a 60× (1.00W) water immersion objective (Olympus). The microscope bath was perfused with oxygenated (95% O_2_, CO_2_ 5%) ACSF consisting of (in mM): 125 NaCl, 3 KCl, 25 glucose, 25 NaHCO_3_, 1.25 NaH_2_PO_4_, 2 CaCl_2_, and 1 MgCl_2_. Based on the myelin structure visualized in the bright-field image large L5 neurons with an intact axon parallel to the slice surface were targeted for simultaneous somatic, somato-dendritic or somato-axonal whole-cell current-clamp recording using Dagan BVC-700A amplifiers (Dagan Corporation, MN, USA). Bridge balance and capacitance were fully compensated based on small current injections leading to minimal voltage error (Figure S1). Voltage was analog low-pass filtered at 10 kHz (Bessel) and digitally sampled at 50 kHz for subthreshold data and 100 kHz for action potentials using an A/D converter (ITC-18, HEKA Elektronik Dr. Schulze GmbH, Germany) and data acquisition software Axograph X (v.1.5.4, Axograph Scientific, NSW, Australia). Patch pipettes were pulled from borosilicate glass (Harvard, Edenbridge, Kent, UK) pulled to an open tip resistance of 5–6 MΩ. The intracellular solution contained (in mM): 130 K-Gluconate, 10 KCl, 4 Mg-ATP, 0.3 Na_2_-GTP, 10 HEPES and 10 Na_2_-phosphocreatine (pH 7.4 adjusted with KOH, 280 mOsmol kg^−1^). Passive membrane responses were collected in the presence of a blocking solution in which 25 mM NaCl was replaced by 20 mM tetraethylammonium (TEA) chloride and 5 mM 4-aminopyridine (4-AP; a non-selective Kv1, Kv2 and Kv3 channel blocker) and by adding to the solution 1 μM tetrodotoxin (TTX) to block sodium channels, 20 μM ZD-7288 to block hyperpolarization-activated cyclic nucleotide-gated (HCN) channels, 10 μM XE-991 for Kv7 (KCNQ) channels and 0.2 mM CdCl_2_ to block voltage-gated calcium channels. To further reduce synaptic depolarizations we added 20 μM of the AMPA receptor blocker 6-cyano-7-nitroquinoxaline-2,3-dione (CNQX).

#### Voltage-sensitive-dye (VSD) imaging

To optically record transaxonal *V*_m_, large L5 pyramidal neurons were labeled with a membrane-embedded red voltage-sensitive dye JPW3028 delivered via somatic patch pipettes in the whole-cell configuration (Hamada et al., 2017, Popovic et al., 2011). Its close analog JPW1114 is characterized by the same voltage sensitivity and is commercially available (D6923, ThermoFisher Scientific). Patch pipettes were first filled with dye-free solution for half of the tapered part of the pipette tip, then backfilled with the dye-containing solution (0.8 mM JPW3028). Intracellular filling was performed for 0.5–1 hr followed by an additional 1.5–2 hr at room temperature without pipette. The soma was re-patched for electrical stimulation and optical recording using a patch pipette filled with standard dye-free intracellular solution. For voltage imaging, a stationary upright microscope (Olympus BX51WI, Olympus, Japan) was equipped with two camera ports. One for high spatial resolution with a CCD camera for oblique contrast video-microscopy (CoolSNAP EZ, Photometrics) and the other port had a fast data-acquisition camera (up to 20 kHz) with relatively low spatial resolution (80 × 80 pixels) but high dynamic range (14 bits) and low read noise (NeuroCCD-SM, RedShirtImaging LLC, Decatur, GA). The slice was placed on the stage of the microscope and the fluorescent image of the stained neuron was projected by a water immersion objective (either 60× /1.0 NA, Olympus, Japan or 100× /1.1 NA, Nikon, Japan) onto the fast data-acquisition CCD positioned in the primary image plane. Optical recordings were obtained with wide-field epifluorescence microscopy. A frequency-doubled 500 mW diode-pumped Nd:YVO4 continuous wave laser emitting at 532 nm (MLL532, Changchun New Industries Optoelectronics Tech. Co., Ltd., Changchun, China) was the source of excitation light. The laser beam was directed to a light guide coupled to the microscope via a single-port epifluorescence condenser designed to provide approximately uniform illumination of the object plane (adapted from an X-cite lamp, Burleigh, Canada). The fractional noise of the solid-state lasers (RMS < 0.5%) is below typical fractional shot-noise in fluorescence voltage-sensitive dye recordings to maximize the sensitivity of *V*_M_ imaging. This was achieved via a monochromatic excitation light at the red wing of the absorption spectrum. For calibrated AP measurements (Figure 6), the excitation light was reflected to the preparation by a dichroic mirror with a central wavelength of 560 nm, and the fluorescence light was passed through a 610 nm barrier filter (adapted from Olympus U-MWG filter assembly cube). The image of a stained neuron was projected onto a CCD chip via a 0.1× demagnifier. In combination with the 100× objective our CCD frame (80 × 80 pixels) corresponded to pixels receiving light from an area between ∼2 to 6 μm^2^ (with 10 – 20 kHz acquisition frame rate, respectively). The center of internodal regions was on average at 37.2 ± 2.5 μm (n = 12) distance from the node. For optical recording of passive voltage transients (Figure 5) the excitation light was further optimized by reflecting it to the preparation via a dichroic mirror designed specifically to reflect 532 nm laser line and pass the wavelengths between 541.6 and 1200 nm with 93% efficiency. Furthermore, a 532 nm notch filter, designed to remove all traces of the laser excitation, was added to the light path. The image of a stained neuron was projected onto a CCD chip via a 0.37× demagnifier. In combination with the 100× objective our CCD frame (80 × 80 pixels) corresponded to pixels receiving light from an area of ∼0.76 μm^2^ (with 2 kHz acquisition frame rate). Since our optical measurements were based on a comparison of VSD signals recorded from different locations, light intensity must be linearly proportional to *V*_m_ over the entire voltage range. This has been demonstrated repeatedly with JPW3028 (equivalent to JPW1114); in particular, its ability to accurately track the full-size action potential in L5 pyramidal neurons (Popovic et al., 2011). To convert fluorescence into absolute *V*_m_ a calibration protocol was developed based on experimentally recorded steady-state voltage attenuation in L5 myelinated axons (Figure S5).

#### Histochemistry and immunofluorescence

For morphological reconstruction and/or nodal staining 5 mg ml^−1^ biocytin and/or 100 μM Alexa Fluor salts (488 or 594) were added to the intracellular solution. The 300 μm thick slices were rinsed in 0.1 M PBS and quenched in 3% H_2_O_2_ twice for 30 min. Following increasing washing steps in fresh 0.1 M PBS sections were incubated in series of increasing sucrose solutions (10%, 20% and 30% in PBS for 45 minutes). The membrane was permeabilized by several cycles of rapid liquid nitrogen freezing and thawing after which they were incubated in 1% avidin-biotinylated horseradish peroxidase H complex (Vector Laboratories) overnight at 4°C. The peroxidase was localized with 0.05% 3,3′-diaminobenzidine peroxidase substrate chromogen for visualization. Slices were washed and mounted in Mowiol. Neuronal morphology was fully reconstructed, including axonal and dendritic branch lengths and diameters, using a 63× oil-immersion objective (numerical aperture *NA* of 1.4, Zeiss) coupled with the 3D tracing software Neurolucida (v.11, MicroBrightField Europe, Magdeburg, Germany). Consistent with previous observations (Kole et al., 2007), when comparing axon lengths based on the bright-field image during the physiological recording in slices with the final 3D reconstructions from the same cell, the shrinkage was found to be minimal in the *x-* and *y-*direction (< 2%, n = 8). No correction factor was therefore applied to the neuronal reconstructions. The locations and dimensions of nodes of Ranvier are well visible as an increased intensity of the biocytin-DAB signal or presence of collaterals (Figures S2 and S3). To confirm the locations of reconstructed nodes of Ranvier (Figure S2) using immunofluorescence (Figure S3), L5 neurons (n = 8) were filled with biocytin and fixed for 20 minutes in 4% paraformaldehyde (PFA), then stored in 0.1 M PBS (pH 7.4). Sections were blocked in 5% normal goat serum (NGS) followed by 24 h incubation in primary antibodies diluted in 0.1 M PBS containing 5% NGS, and 2% Triton X-100. Sections were stained with rabbit anti-βIV-spectrin (1:200, gift from M. N. Rasband, Baylor College of Medicine, TX) and biocytin was visualized using Streptavidin-488. Sections were mounted and examined with confocal microscopy.

#### Electron microscopy

Four of the six biocytin-filled L5 neurons used for modeling were recovered for EM. Coverslips were carefully removed and the object glass with the section was placed in milliQ water for several days. Afterward, sections were rinsed 0.1 M sodium cacodylate buffer pH 7.4 for a few hours and placed in a 1% osmium tetraoxide solution containing 1.5% potassium ferricyanide in 0.1 M sodium cacodylate buffer pH 7.4 for 10–15 mins. Sections were subsequently dehydrated in a sequence of ethanol dilutions, pure acetone and an acetone/epoxy resin mixture for 30-35 mins, followed by 30 mins in pure epoxy. Slices were then embedded between thin sheets of polymer and sealed at 60°C. For three L5 neurons filled with horseradish peroxidase (HRP) brain slices were fixed in 4% paraformaldehyde (PFA) and/or 5% glutaraldehyde and rinsed thereafter in 0.1 M sodium cacodylate buffer at pH 7.4. Slices were placed in a 25% sucrose solution in 0.1 M sodium cacodylate buffer (pH 7.4). When the slices were saturated, they were embedded in Tissue-Tek in an aluminum boat and frozen by dry ice. Sections were re-cut at 40 μm and subsequently rinsed in a Tris-HCl buffer (pH 7.4). To visualize the peroxidase, the sections were incubated in a Tris–HCl diaminobenzidine (DAB) solution containing 0.03% H_2_O_2_. The DAB reaction product was then intensified by a gold-substituted silver peroxidase. Sections were rinsed in a sodium cacodylate buffer of 0.1 M (pH 7.4) and post-fixed for 20 min in 1% OsO_4_ supplemented with 1.5% potassium ferricyanide in a sodium cacodylate buffer of 0.1 M (pH 7.4). The material was subsequently dehydrated and embedded in epoxy resin, then cut in ultrathin sections. All ultrathin sections were examined and photographed with a FEI Tecnai G12 electron microscope (FEI, Europe NanoPort, Eindhoven, the Netherlands). Images were saved in tiff format and analyzed using Fiji (ImageJ) graphic software (Schindelin et al., 2012)(v1.47p, NIH, USA).

#### High-pressure freeze electron microscopy

To prevent artifacts with aldehyde fixation methods such as splitting, loosening or shrinking of intraperiod spaces in the myelin sheath we used HPF EM (Möbius et al., 2016) to examine the periaxonal width dimensions. Wistar rats (P80–90) were anesthetized and terminated by cervical dislocation. The brain was removed quickly and then cut with a Leica vibratome VT1200S in 200 μm sections. The cortex was cryofixed in 20% poly(vinyl-pyrrolidinone) (Sigma-Aldrich, Munich, Germany) using the high pressure freezer Leica HPM100 (Leica, Vienna, Austria). The freeze-substitution and the subsequent Epon-embedding of the tissue was carried out as described previously (Möbius et al., 2010, Snaidero et al., 2014) using the Leica AFS II. The Epon embedded tissue was cut with the Leica Ultracut S ultramicrotome in 500 nm semithin sections or in 50 nm ultrathin sections that were contrasted with 4% uranylacetate (SPI-Chem, West Chester, USA) (Möbius et al., 2010). Electron micrographs were obtained with the electron microscope LEO EM912AB (Zeiss, Oberkochen, Germany) equipped with an on-axis 2k CCD-camera (TRS, Moorenweis, Germany) using the ITEM (Olympus, Münster, Germany) software. For quantification of the periaxonal width, only the intercellular distance between clearly cross-sectioned outer axonal and myelin membranes was measured. Between 51 and 73 measurements were made from 20 images per animal (n = 3).

#### Computational Modeling

Electrophysiological recordings were combined with reconstructed morphologies in the NEURON simulation environment (v.7.3–7.5) (Hines and Carnevale, 2001) and custom-written software. Neuron morphologies were uploaded to NEURON via its Import3D tool. Nodal and internodal domains were incorporated into the morphological reconstructions as described above. Paranodal domains were implemented post hoc with a fixed length of 2.3 μm, based on our longitudinal EM data and consistent with previous estimates in mammalian central nervous system (Shepherd et al., 2012). An evolutionary algorithm was developed to robustly search the parameter space of each circuit model, running for a total modeling time of over 3 million core hours on the Comet and Stampede2 supercomputers at the Neuroscience Gateway (Sivagnanam et al., 2013). The massively parallel computational approach enabled each parameter space to be thoroughly and unbiasedly searched, and resulted in thousands of unique solutions for each circuit implementation, with the unbiased selection of the lowest error solution representing each simulation. To substantially reduce runtimes (up to 50%), DC models were run in a recompiled version of NEURON to the necessary 1 extracellular layer instead of the default 2 (currently available at the Neuroscience Gateway), allowing for the combined modeling of *V*_m_ and *V*_my_ (v and vext[0], respectively, in the single layer extracellular version of NEURON).

##### Spatial Resolution and Parameter Bounds

Spatial resolution was set by the d_λ_ rule for optimal computational efficiency (Hines and Carnevale, 2001). To ensure sufficient spatial resolution within the d_λ_ rule, a minimal τ_m_ was used to compute *f*_m_, to maximize the number of segments per cellular compartment. Minimal τ_m_ was approximately 1 ms, determined by direct exponential fit to averaged axonal responses with noisy traces removed (see Uncertainty Analysis below). Parameter bounds are listed in Table S1. The lower bound for *R*_my_ was based on an expected minimum of 10 myelin membranes at the lower bound for a single myelin membrane (Bakiri et al., 2011, Chan et al., 2013). The upper bound for *R*_my_ was based on Equations 7 and 8, with an expected maximum of 100 myelin membranes at the upper bound for a single myelin membrane (Bakiri et al., 2011). The lower bound for *C*_my_ was based on Equations 9 and 10, with an expected maximum of 100 myelin membranes at the lower bound for *C*_m_. The upper bound for *C*_my_ was based on Equations 9 and 10, with an expected minimum of 10 myelin membranes at the upper bound for *C*_m_. To allow *r*_pa_ to represent approaching extremely large δ_pa_, the lower bound for *r*_pa_ was based on a *R*_pa_ of 35 Ω cm, the intracellular resistivity of the squid giant axon (Hodgkin and Huxley, 1952), minimum *d* of 0.5 μm and maximum δ_pa_ of 300 nm (the approximate width of the myelin sheath). To allow *r*_pa_ to represent extremely small δ_pa_, the upper bound was based on the resistivity of deionized water (2 MΩ cm), which basically encompasses a SC version of the double cable. The parameter bounds were within a maximum *d* of 2 μm and minimum δ_pa_ of 1 nm, to stay above the Debye limit of 0.78 nm estimated for extracellular fluid (Hille, 2001). The lower bound for *r*_pn_ was the same as for *r*_pa_, and its upper bound was 100× the upper bound for *r*_pa_, the expected maximum. All optimized cable parameters were given equal weight during optimization.

##### Fit start time

The fit start time for the recordings from injecting/recording electrodes was fixed to 0.5 ms following the end of current injection, to account for lingering effects from uncompensated discharging pipette capacitance artifacts in the voltage response. The fit start time of +0.5 ms was estimated from dual somatic recordings in which one recording pipette injected current and recorded the voltage response, creating a voltage artifact, and the other pipette was a voltage follower only (Figure S1). Comparison of the voltage traces from each pipette revealed that the time between current injection end and resumption of maximum trace overlap from each electrode was approximately +0.5 ms post-injection (0.466 ± 0.0403 ms, n = 24 traces from 3 cells across 8 different current injection recordings: ± 300, ± 400, ± 500 and ± 600 pA). An optimization start time of +0.5 ms following current injection in injecting/recording electrode responses was thus implemented across all simulations.

##### Inclusion of recording pipettes

Glass pipette tips were explicitly modeled and connected to model cells at their recording location, to parse out their biophysical contributions to cellular properties (Major et al., 1994, Nörenberg et al., 2010, Roth and Häusser, 2001, Schmidt-Hieber et al., 2007). Tips were built from 200 cylindrical sections, each 10 μm in length, growing in diameter from 1 μm (based on bright-field imaging) to 530 μm, as only the first 2 mm of the tip was modeled, the same length as that of the longest model cell (cell #4, axo-apical axis). The axial resistivity of the pipette was set by the bridge balance of the corresponding electrode (Roth and Häusser, 2001), defined as the ratio of the product of bridge balance and total pipette length (0.2 cm) to the sum of the cross-sectional area of each section (Nörenberg et al., 2010). Given the upper and lower bound for *R*_my_ and *C*_my_ (Table S1), the specific pipette wall resistance and capacitance were assessed, to ensure these did not mitigate the values of *R*_my_ and *C*_my_. The specific resistance of the pipette wall was calculated from the trans-resistivity of the pipette composite material (Harvard Apparatus GC150F 300060) estimated at 100 MΩ cm (Ehrt, 2009). Based on an initial wall thickness of 0.5 μm at the tip and pipette lateral surface area (simplified to a truncated cone), the specific wall resistance was calculated and fixed to 50 GΩ cm^2^, a value only 30× bigger than the upper bound for *R*_my_. Input pipette capacitance (*C*_pip_) was optimized in short passive responses from injecting/recording electrodes at the soma or dendrites, to parse out fluctuating compensated *C*_pip_ values from *C*_m_ and particularly *C*_my_, since the upper bound for *C*_pip_, based on the estimated uncompensated radial capacitance of the modeled pipette tip (see below), was only 4× smaller than the lower bound for *C*_my_ (Table S1). We consider lingering but compensated *C*_pip_ values during APs as negligible, due to the much larger capacitive response of the cell. The modeled pipette is essentially a tapering cylinder with inner and outer radii at the front tip (*r*_inner_ and *r*_outer_) and back tip (*R*_inner_ and *R*_outer_). The front end and horizontal plane form an angle α, together defining *C*_pip_ as (Wang et al., 2013):

(Equation 1) $$C_{pip}=\frac{2\pi L_{pip}K_{pip}\varepsilon_{0}}{\sin\left(\alpha \right)\ln\left[\frac{2r_{outer}+L_{pip}\;\cot\left(\alpha \right)}{2r_{inner}+L_{pip}\;\cot\left(\alpha \right)}\right]}$$

The dielectric constant of the pipette material *K*_pip_ was 4.7 (Harvard Apparatus GC150F 300060). *L*_pip_ represented the length of the pipette and ε_0_ the permittivity constant of free space (8.85 × 10^−8^ μF cm^–1^). The definition of *A*_pip_, the outer lateral surface area of the modeled pipette tip follows as:

(Equation 2) $$A_{pip}=\pi \left(R_{outer}+r_{outer}\right)\sqrt{{\left(R_{outer}-r_{outer}\right)}^{2}+L_{pip}^{2}}$$

Combining Equations 1 and 2, the fully uncompensated value for *C*_pip_ was 129 pF cm^–2^, a value only ∼40× smaller than the lower bound for *C*_my_ (Table S1) The average stray uncompensated *C*_pip_ value of injecting/recording pipettes in the DC model cells (n = 6) was 5.52 × 10^−5^ μF cm^–2^, a value ∼700× smaller than *C*_my_ (Table S4).

#### Modeling approach and sensitivity analysis

An evolutionary algorithm for model optimizations was employed for the following reasons: 1) to find with an unsupervised approach the lowest available error within the solution search-space; 2) to compare multiple circuit-specific solutions statistically to a randomized one for parameter, as well as direct circuit-to-circuit, sensitivity analysis and 3) to solve the initial parameter value sensitivity problem by stochastically canvasing the initial value space. 1) was achieved via 3) and a modified optimization procedure embedded within the evolutionary algorithm, based on Brent’s PRAXIS method (Brent, 1973), which is built into NEURON. Briefly, our optimization procedure addresses the problem of non-uniformly distributed parameters, such as myelin parameters, being less constrained than uniformly distributed ones, such as cellular membrane *C*_m_ (over an entire cell), by constraining optimization to experimental noise. Essentially, optimization was forced to exit if error improvement was marginal and within experimental noise (defined as signal variance over the delay to current injection). This approach led to greater simulation efficiency, producing runtimes orders of magnitude faster than the default search routine. To further maximize PRAXIS efficiency, parameters were normalized and log transformed. The randomized control trial mentioned in 2) was the 0 pA current injection in each cell, used for that cell’s results from the different current injection trials used (Cells #1-6, ± 300, ± 400, ± 500 and ± 600 pA). Circuit sensitivity analysis, comparing the single and double cable models, revealed *R*_my_ and *C*_my_ well constrained relative to control (three-way ANOVA, p < 0.0001; 66%–71% of variation accounted for by optimized versus control alone or with cell # versus optimized versus control; 16%–22% by single versus double cable; 256–1024 evolution-optimized solutions compared for each cell; n = 6 cells). Periaxonal resistance, specific only to the double cable circuit, was highly sensitive relative to control, indicating this parameter was well constrained by the electrophysiological recordings (two-way ANOVA p < 0.0001, 70% of variation accounted for by cell # and injection amplitude; Bonferroni comparisons test between cells p < 0.01 for cell #1 and p < 0.0001 for Cells #2-6).

#### Uncertainty analysis

Multiple sources of systemic error that could influence the modeling were addressed. Sampling error was addressed by gathering between 30 and 180 voltage responses per injection level per cell. Further, noisy trials were discarded if more than 5% lay outside of two standard deviations from the mean for that current injection level. To account for false-positive identification of a noisy trial, the duration of a noisy data point needed to have a minimum temporal resolution above the Bessel filter cut-off applied at the amplifier (10 kHz). For the morphological reconstructions, although main axons were well visible and within the resolution of bright-field microscopy, sealed myelin weakens emission signals, possibly leading to error in the 3D morphological tracing of internodal sections. To account for the possibility of such measurement error, the resolving distance for the morphological reconstructions was determined according to the microscope setup. Given the numerical apertures of the microscope objective and condenser, the resolving distance *(rd*) was calculated as follows:

(Equation 3) $$rd=\frac{1.22\lambda }{NA_{obj}+NA_{cond}}$$

Given *NA*_obj_ = 1.4, *NA*_cond_ = 0.9 and λ = 550 nm, the *rd* was approximately 300 nm. A random factor of 300 nm (random normal distribution of mean = 0 and variance = 1) was thus added or subtracted from each discrete diameter measurement of a given model axon internode, and re-optimized according to single or double cable circuit implementations (Figure 1). 32–128 simulations were performed for each circuit in each cell for each current injection (n = 6 neurons with 8 voltage responses optimized from 8 current injections each with a single or double cable circuit; 7680 simulations total). The double cable model maintained a consistently lower fit error at the individual somatic or axonal transient level (soma or axon: paired t test p < 0.0001 or p < 0.0001, respectively; n = 6 neurons). To address the effectiveness of voltage-gated and synaptic channel blockers in the myelinated axon, other possible sources of current leakage within the myelin sheath, and the influence of additional cable parameters in either SC or DC circuit implementations, a SC with a separate node of Ranvier membrane resistance (*R*_mN_), separate internodal axolemma membrane resistance (*R*_mI_), separate nodal and internodal axolemma membrane resistances (*R*_mN_ and *R*_mI_), and separate myelin sheath resistance and capacitance for each internode present, from 1 to 6 internodes (*R*_my1_,…,*R*_my6_ and *C*_my1_,…,*C*_my6_), was optimized in the same evolutionary paradigm. All alternative SC circuits had a combined axo-somatic fit error equivalent or greater than that of the basic SC (Friedman test with Dunn’s correction p > 0.245–0.999). Moreover, all SC models had much higher combined errors than the basic DC model (with *r*_pa_ throughout the internode, including the paranodes; Friedman test with Dunn’s correction p < 0.0001). A direct comparison of the basic DC model (*r*_pa_ distributed throughout the internode without a separate *r*_pn_ at the paranodes), to the DC model (with a separate *r*_pn_ at the paranodes), demonstrated a lower fit error for the DC model over the entire axonal passive voltage response, and the poorer fit was particularly visible at the highly non-uniform rise times and decay phases (whole-trace paired t test: axonal p < 0.0465, somatic p > 0.233; n = 6 neurons). To ensure the distinction between SC and DC models lay in the internode, we compared the *R*_i_, *R*_m_ and *C*_m_ obtained in dual somato-somatic, somato-dendritic and somato-AIS recordings in model cells without myelin parameters in their reconstructed model axons, with those from axo-somatic recordings (SC and DC; Figures 1 and S1, Tables S2–S4). We recorded from 6 cells, the same as in the axo-somatic recordings, and found the same *R*_i_, *R*_m_ and *C*_m_ across circuits (ordinary one-way ANOVA with Bonferroni’s correction; *R*_i_: p > 0.255–0.999; *R*_m_: p > 0.999; *C*_m_: p > 0.583–0.999; n = 6 cells).

#### Cellular Equations

Axial resistance is the ratio between axial resistivity and cross-sectional area. In the case of the axon core, treated as a cylinder, axial resistance (*r*_i_) is defined as (Rall, 1977):

(Equation 4) $$r_{i}\;=\;\frac{4R_{i}}{\pi \;d^{2}}$$

In Figure 3, *r*_i_ is taken from internodal axon only, utilizing optimal *R*_i_ and given internodal *d* (Table S4). Axial resistance in the periaxonal space (*r*_pa_) is similarly defined as *r*_i_ as the ratio between periaxonal resistivity (*R*_pa_) and periaxonal cross-sectional area, where the axon core cylinder is surrounded by the larger cylinder which includes the periaxonal space. This larger cylinder is of diameter *d* + 2δ_pa_, where δ_pa_ is the radius of the periaxonal space, yielding the following relationship between *r*_pa_ and *R*_pa_ (Halter and Clark, 1991):

(Equation 5) $$r_{pa}\;=\;\frac{R_{pa}}{\pi \;\delta_{pa}\;\left(d+\delta_{pa}\right)}$$

For calculating *R*_pn_, δ_pn_ was fixed at 7.4 nm (Nans et al., 2011). Similarly, δ_pa_ (or δ_pn_) can be predicted by isolating δ in Equation 5:

(Equation 6) $$\delta_{pa}\;=\;\frac{1}{2}\left[-d+\sqrt{d^{2}+\left(\frac{4\;R_{pa}}{\pi \;r_{pa}}\right)}\right]\;$$

To predict or vary the number of myelin lamellae (*n*_my_) in a given single or double cable axon model, the two electrical parameters defining the sheath (*R*_my_ and *C*_my_) were manipulated simultaneously. Recognizing that a myelin sheath is an in-series compaction of *n* layers, the radial resistance of the sheath (*R*_my_) is the sum of the resistances of each myelin membrane (*R*_mm_):

(Equation 7) $$R_{my}=\sum_{i=1}^{n}R_{mm_{i}}$$

Assuming each myelin membrane is electrically identical, the resistance of a single myelin membrane (*R*_mm_) may thus be expressed as a function of the number of myelin lamellae (*n*_my_):

(Equation 8) $$R_{my}=2n_{my}R_{mm}$$

Similar to Equation 7, the radial capacitance of the myelin sheath (*C*_my_) may be defined according to the sum of the capacitances of each of its composing membranes (*C*_mm_):

(Equation 9) $$\frac{1}{C_{my}}=\sum_{i=1}^{n}\frac{1}{C_{mm_{i}}}$$

Similar to Equation 8, with each myelin membrane considered as electrically identical, *n*_my_ can separately be expressed as a function of the capacitance of a single myelin membrane (*C*_mm_):

(Equation 10) $$C_{my}=\frac{C_{mm}}{2n_{my}}$$

To predict an average *n*_my_ from the double cable modeling results (Table S4), Equation 10 was applied with average *C*_m_ and *C*_my_ values (Figure 2D). To separately predict values for *R*_mm_ and *C*_mm_, Equations 8 and 10 were applied with the average *n*_my_ measured by EM combined with each optimized value for *R*_my_ and *C*_my_, comparing these with each corresponding optimized value for *R*_m_ and *C*_m_.

To predict δ_my_, the radius of the myelin sheath, each myelin membrane may be considered as a separate slice of the myelin sheath capacitor with a given radius δ_mm_. If each repeated layer possesses the same dielectric constant (κ_my_), and the permittivity of each layer (ε_my_) is the product of κ_my_ and ε_0_, the sum of the radius of each layer yields:

(Equation 11) $$C_{my}=\varepsilon_{my}\sum_{i=1}^{n}\frac{1}{\delta_{my_{i}}}$$

If κ_my_ is the same as the dielectric constant of internodal axolemma (κ_m_), then the following relation emerges:

(Equation 12) $$\delta_{m}C_{m}=\delta_{my}C_{my}$$

δ_my_ can be predicted by fixing δ_m_ to 8 nm, a typical thickness for cellular membrane that includes its constituent proteins (Singer and Nicolson, 1972).

#### Active conductance-based double cable model

For conductance-based multi-compartmental simulations we used cells #3 and #6 (Figure S2), fixing their cell-specific optimized passive cable parameters *R*_i_, *R*_m_, *C*_m_, *r*_pa_, *r*_pn_, *R*_my_ and *C*_my_ (Table S4) and implementing standard models for voltage-gated conductances. The initial potential of the model was set to obtain the recorded resting membrane potential (approximately –75 mV, taking into account the –12 mV junction potential), and simulation temperature was set to that of the recording (∼35°C). We added Nav conductance by implementing two separate 8-state allosteric sodium channel models developed for the soma and axon (Schmidt-Hieber and Bischofberger, 2010). In addition, we added conductances in the AIS, soma and dendrites for HCN, voltage-gated K^+^ conductance models (Kv1, Kv2/3 and Kv7) and calcium-dependent K^+^ models, based on previous L5 pyramidal neurons (Battefeld et al., 2014, Hallermann et al., 2012, Hamada et al., 2016). Following manual optimization, we used an unsupervised optimization routine similar to that used for the passive transients to simultaneously optimize both somatic and axonal APs. In the absence of knowledge about K^+^ diffusion dynamics within internodes we did not compartmentalize juxtaparanodal domains and implemented Kv1 channels into the node of Ranvier to support rapid repolarization. The optimized distribution of the optimized peak conductance densities for cell #3 with which Figure 7 was generated was as follows (all in pS μm^–2^); the AIS contained Nav, Kv1 and Kv7 channel densities at (40.000, 1000 and 200), the soma had Nav, Kv1 and Kv7 channels (650, 100 and 20). Dendritic compartments contained a distance-dependent gradient of Nav channel densities (from 600 to 40 in the distal dendrites), a uniform density of Kv1 and Kv7 (22 and 30, respectively). The internodes had low densities of HCN, Nav, Kv1 and Kv7 channels (1, 2, 2, and 0.1 respectively). Nodes of Ranvier contained high densities of Nav, Kv1 and Kv7 channel (45.000, 1000 and 100, respectively). The unmyelinated cut end had modest densities Nav, Kv1 and Kv7 channels (525, 380 and 1, respectively). The K^+^ and Na^+^ equilibrium potentials were set to –85 and +55 mV, respectively.

### Quantification and Statistical Analysis

30 to 180 trials were recorded per current injection amplitude for the brief current pulses in passive conditions (2 ms step duration, 100 ms total). Traces were first baselined by subtracting their average voltage over 70% of the delay time preceding the pulse. For the VSD optical recordings, 100 trials were performed for each cell. Individual responses for each location were aligned and averaged. Onset latency was defined as the delay to half-maximum amplitude. Conduction velocity was defined according to the onset time of the first rapidly changing phase of the AP, i.e., its voltage threshold (Kole and Stuart, 2008). For recorded APs, threshold onset was defined as a voltage rate-of-rise minimum in d*V*/dt greater than 3× the standard deviation of the recording noise (initial, non-current-injected part of the voltage response), and ranged from 60–120 *V* s^–1^. For model APs, threshold onset was defined directly as occurrence of the first peak in d^3^*V*/dt^3^. The distance used in determining conduction velocity was the path length between the two given locations, determined by morphological reconstruction and NEURON’s distance function. All data plotting and statistical analyses were performed in Prism (version 7, GraphPad), Igor Pro (version 6.37, WaveMetrics) or SPSS (version 23, IBM Analytics). Details of the statistical analyses, including tests, representation and value of *n*, center, dispersion and precision measures can be found in corresponding figure legends. Normality (D’Agostino Pearson omnibus) was tested wherever possible to justify the use of parametric tests; otherwise non-parametric tests were used.

### Data and Code Availability

The source data for Figure 1 in the paper and alternative circuit implementations is available on Mendeley (https://data.mendeley.com/) via https://doi.org/10.17632/xkh45t8dmm.1. All custom software for data processing, analysis, automated optimization and model comparisons are available from GitHub (https://github.com/Kolelab). The NEURON model of cell #6 with a double cable implementation of myelin to generate Figure 7 and Videos S3 and S4 in this paper can be downloaded from ModelDB (https://modeldb.yale.edu/260967).
