# Supplementary material for: Saltatory Conduction along Myelinated Axons Involves a Periaxonal Nanocircuit
Source: Cell. 2020 Jan 23;180(2):311–322.e15. doi: 10.1016/j.cell.2019.11.039 (PMC6978798; doi:10.1016/j.cell.2019.11.039)
Supplement: Document S1. Tables S1–S4 [file mmc1.pdf]

Cell, Volume *180*

Supplemental Information

Saltatory Conduction along Myelinated

Axons Involves a Periaxonal Nanocircuit

Charles C.H. Cohen, Marko A. Popovic, Jan Klooster, Marie-Theres Weil, Wiebke Möbius, Klaus-Armin Nave, and Maarten H.P. Kole

Table S1, related to Figure 1 | Boundaries of the cable parameter solution space

Parameter	Lower Bound	Upper Bound
R_i (Ω cm)	50 [a]	400 [b]
R_m (kΩ cm^2)	0.1 [c]	200 [b]
C_m (μF cm^{-2})	0.5 [d]	2 [e]
R_{my} (kΩ cm^2)	20 [f]	1600 [f]
C_{my} (μF cm^{-2})	0.005	0.2
r_{pa} (GΩ cm^{-1}) [g]	5	2.5×10^7
r_{pn} (TΩ cm^{-1}) [g]	0.05	2.5×10^6

[a](Fenstermacher et al., 1970). [b](Major et al., 1994). [c](Rapp et al., 1994; Tasaki, 1955). [d](Major et al., 1994). [e](Rapp et al., 1994). [f](Bakiri et al., 2011; Chan et al., 2013). [g]The r_{pa} and r_{pn} parameter apply to double cable models only.

Table S2, related to Figure 1 | Optimal cable parameters for single cable models

Cell (#)	R_i (Ω cm)	R_m (kΩ cm^2)	C_m (μF cm^{-2})	R_{my} (kΩ cm^2)	C_{my} (μF cm^{-2})
1	93.1	22.9	0.953	50.5	0.185
2	134	23.9	1.08	1180	0.154
3	376	23.3	1.77	37.6	0.163
4	243	23.3	1.24	73.0	0.189
5	211	23.5	1.19	991	0.187
6	144	22.0	1.45	842	0.166
**Average**	**200**	**23.1**	**1.28**	**530**	**0.174**
**SEM**	**35.2**	**0.218**	**0.101**	**184**	**0.00510**

Cable parameter solutions for neuronal (R_i, R_m and C_m) and myelin sheath parameters (R_{my} and C_{my}).

Table S3, related to Figure 1 | Optimal cable parameters for somato-somatic, somato-dendritic, and somato-AIS recordings

Cell (#)	V_{inj} location	V_{rec} location	R_i (Ω cm)	R_m (kΩ cm^2)	C_m (μF cm^{-2})
7	Soma	Soma	155	19.1	1.31
8	Soma	Soma	111	21.9	1.37
9	Soma	Soma	83.9	21.5	0.902
10	Dend	Soma	139	31.1	1.18
11	Soma	Dend	150	24.5	1.01
12	AIS	Soma	75.3	29.4	0.838
**Average**			**119**	**24.6**	**1.10**
**SEM**			**14.0**	**1.94**	**0.0893**

Table S4, related to Figure 1 | Optimal electrical parameters for double cable models

Cell (#)	R_i (Ω cm)	R_m (kΩ cm^2)	C_m (μF cm^{-2})	r_{pa} (GΩ cm^{-1})	r_{pn} (TΩ cm^{-1})	R_{my} (kΩ cm^2)	C_{my} (μF cm^{-2})
1	230	18.9	1.23	84.4	1.50	467	0.0118
2	98.3	25.2	0.986	135	4.39	101	0.0115
3	261	25.2	1.37	141	3.56	579	0.00769
4	158	25.8	1.06	188	4.07	152	0.0161
5	109	27.7	1.01	105	0.877	75.2	0.0678
6	71.2	24.8	1.23	96.3	0.321	63.7	0.113
**Average**	**155**	**24.6**	**1.15**	**125**	**2.45**	**240**	**0.0379**
**SEM**	**31.2**	**1.22**	**0.0617**	**0.155**	**0.719**	**91.6**	**0.0175**

Cable parameter solutions for neuronal (R_i, R_m and C_m), submyelin (r_{pa} and r_{pn}) and myelin sheath parameters (R_{my} and C_{my}). The morphologies of cell#1 to #6 are shown in Figure S2.
